# Transcriptome Analysis of *Populus tomentosa* Reveals Molecular Mechanisms of Jasmonate Signaling Pathway on Arsenic Tolerance Induced by Arbuscular Mycorrhizal Fungi

**DOI:** 10.3390/biology15181653

**Published:** 2026-09-18

**Authors:** Qiaoming Zhang, Wenjing Yang, Jiajie Su, Wenya Lv, Lei Wang, Shanshan Xu, Qingshan Chang, Minggui Gong

**Affiliations:** 1 College of Horticulture and Plant Protection, Henan University of Science and Technology, Luoyang 471023, China; zhangqm1013@163.com (Q.Z.); ywenjing@126.com (W.Y.);; 2 College of Food and Bioengineering, Henan University of Science and Technology, Luoyang 471023, China17839917264@163.com (W.L.)

**Keywords:** *Populus tomentosa*, arbuscular mycorrhizal fungi, arsenic stress, jasmonate signaling pathway, transcriptome

## Abstract

Arsenic (As) pollution poses serious threats to soil ecosystems and plant growth. Arbuscular mycorrhizal fungi (AMF) have been confirmed to enhance As tolerance in host plants, making mycorrhizal-assisted phytoremediation a promising and practical strategy for remediating As-contaminated soils. *Populus tomentosa* Carr. (Chinese white poplar) is a native fast-growing woody species suitable for phytoremediation in East Asia. However, comprehensive transcriptomic analyses focusing on the molecular mechanisms by which AMF improve As tolerance in this species remain limited. In this study, a pot-based experiment was performed on *P. tomentosa* seedlings using a two-factor experimental design with four treatments: non-inoculated seedlings under non-As stress (CK0), non-inoculated *P. tomentosa* seedlings under As stress (CK100), *Rhizophagus irregularis*-inoculated seedlings under non-As stress (Ri0), and *R. irregularis*-inoculated seedlings under As stress (Ri100). Plant-growth measurements, root morphological assessment, and Illumina RNA-seq transcriptomic analysis were applied to characterize seedling responses. Our results revealed that As stress significantly inhibited AMF colonization rate, suppressed seedling growth, and disrupted root morphological architecture. Nevertheless, *R. irregularis* inoculation substantially alleviated As-induced growth repression, increasing plant height, shoot and root dry biomass, as well as key root morphological parameters under As exposure. Transcriptome profiling identified large sets of differentially expressed genes (DEGs) triggered by AMF symbiosis and As stress. Functional enrichment indicated that signal transduction of jasmonate (JA) biosynthesis and metabolism represented the dominant response pathways. AMF symbiosis dynamically rewrote the transcriptional patterns of core JA biosynthesis and metabolism genes in *P. tomentosa* seedlings under As stress. Weighted gene co-expression network analysis further highlighted hub transcription factors, including GATA5 and WRKY57, which were tightly co-expressed with JA-synthesis-related genes and potentially bridged mycorrhizal symbiotic signals and downstream defense responses. These findings illustrated that AMF enhanced As tolerance in *P. tomentosa* seedlings by reprogramming JA-associated transcriptional regulatory networks. This study provided novel mechanistic insights for understanding AMF-wood plant-As interactions, and offered theoretical support for developing AMF-assisted poplar phytoremediation technology in As-contaminated soils.

## Simple Summary

Arsenic (As) contamination represents a global soil-pollution issue that impairs plant growth and threatens human food safety. Arbuscular mycorrhizal fungi (AMF) can form beneficial symbiosis with plant roots and improve plant resistance to toxic metalloids. *Populus tomentosa*, a fast-growing poplar species, shows great potential for As phytoremediation. However, it is still unclear how AMF mediate As stress resistance in this tree species. In this study, a pot experiment was conducted with or without *Rhizophagus irregularis* inoculation under non-As or As stress conditions. AMF colonization status, plant-growth performance, and root morphological traits were determined, and transcriptome sequencing was conducted. As stress limited AMF colonization and damaged *P. tomentosa* seedling performance, while *R. irregularis* inoculation improved biomass and root development to relieve As toxicity. Transcriptome sequencing revealed that many differentially expressed genes were induced by *R. irregularis* inoculation and As stress. Pathways related to signal transduction of jasmonate (JA) biosynthesis and metabolism were strongly activated by *R. irregularis* inoculation under As stress. Key transcription factor hubs such as GATA5 and WRKY57 linked mycorrhizal signals to JA-related gene expression in *P. tomentosa* seedlings under As stress. Our results revealed that AMF regulated JA-dependent transcriptional networks to enhance As tolerance in *P. tomentosa* seedlings, supporting the application of AMF-assisted poplar phytoremediation for soil remediation.

## 1. Introduction

Arsenic (As) is a toxic and hazardous metalloid widely distributed in the Earth’s crust, and it represents one of the most ubiquitous geochemical and anthropogenic pollutants in the global soil–water–food ecological chain [1,2]. In nature, As is enriched in orpiment, realgar, and various sulfide ores, and it enters the pedosphere through geological weathering, volcanic emissions, and geothermal spring effluents [1]. Over the past century, anthropogenic activities, such as mining and smelting of As-bearing ores, irrigation with As-contaminated groundwater, combustion of fossil fuels, and application of As-containing agrochemicals and phosphate fertilizers, have pushed As concentrations in the pedosphere far beyond geogenic baselines [3,4,5].

Inorganic As exists predominantly as arsenate [As(V)] and arsenite [As(III)], both of which are readily taken up by plant roots via phosphate/arsenate co-transporters and aquaporin-like silicic acid channels [6]. Once inside the plant, As replaces phosphate in ATP and in enzymatic active sites, generates reactive oxygen species (ROS), inhibits photosystem II function, suppresses germination and root elongation, and ultimately leads to growth arrest, biomass loss, even plant death, and vegetation degradation [1,7,8]. The incorporation of As and its inorganic species into the human food chain may trigger severe health consequences, including cancers, cardiovascular disorders, diabetes, impaired immune function, and infertility [1,9]. Traditional physical and chemical remediation approaches for As-contaminated soil, such as soil washing, solidification, and chemical passivation, suffer from high cost, secondary pollution risks, and irreversible soil structural damage, restricting their large-scale field application [10]. By contrast, phytoremediation relying on tolerant woody plants has emerged as an eco-friendly, low-cost, and sustainable restoration strategy for heavy metal-contaminated lands, which can stabilize or extract toxic metalloids while improving soil fertility and ecological landscape simultaneously [11,12,13].

*Populus tomentosa* Carr., a native fast-growing poplar endemic to East China, has demonstrated outstanding advantages for As phytoremediation practice [14]. This species has robust biomass accumulation, extensive fibrous root systems with strong soil penetration capacity, high adaptability to varied soil textures and heavy metal-pollution gradients, and prominent carbon sequestration potential [14,15,16]. The above traits, combined with its stress tolerance, make it a preferred plant for phytoremediation [12,14]. Critically, *P. tomentosa* is a non-edible fiber and timber tree; the risk of As entering the human food chain via poplar is negligible [13]. *P. tomentosa* may survive and grow under moderate As stress, and its root-rhizosphere interface is chemically active enough to modify As speciation and mobility [14]. Nevertheless, the intrinsic As tolerance of *P. tomentosa* seedlings remained limited under severe As pollution, requiring efficient microbial symbiosis strategies to further strengthen its stress resistance and remediation efficiency.

Arbuscular mycorrhizal fungi (AMF), obligate symbiotic soil microorganisms belonging to the Glomeromycota phylum, establish mutualistic symbiosis with over 80% of terrestrial plant species, including poplars [10,17,18,19]. The fungi colonize root cortical cells, forming highly branched arbuscules for nutrient exchange, together with hyphal coils and vesicles [9]. Extraradical hyphae extend several centimeters beyond the root-depletion zone into the bulk soil, greatly expanding the effective absorptive surface area for host plants [20]. In the AMF-plant symbiosis, the plant provides the fungus with 5–15% of its photosynthate (lipids and sugars), while the fungi reciprocate by delivering mineral nutrients—especially phosphorus (P), but also N, K, Zn, Cu, Mg, and others—from soil to plant roots [18,21]. AMF symbiosis is also well-documented to enhance plant tolerance to a suite of abiotic stresses, including drought, salinity, heavy metals (Cd, Pb, and Zn), and metalloids (As) [9,22,23]. Extraradical AMF hyphae immobilize As ions inside fungal cell walls and glomalin-related soil proteins (GRSPs) to reduce plant As uptake [24]. AMF symbiosis also improves root morphological architecture to enhance water and mineral nutrient acquisition [25]. Symbiotic signals activate host antioxidant, osmotic adjustment and ion homeostasis pathways to restore As-induced physiological damage [17].

Jasmonates (JAs, including jasmonic acid, JA; methyl jasmonate, MeJA; and bioactive jasmonoyl-isoleucine, JA-Ile) are lipid-derived plant-specific phytohormones that act as master signal integrators coordinating plant growth, secondary metabolism, developmental programming and stress defense responses [26,27]. The JA signal transduction module has been systematically elucidated in the model plant *Arabidopsis thaliana* and has exhibited high evolutionary conservation across woody fruit trees, forest trees and herbaceous crops [28,29]. Under low intracellular JA-Ile concentration, JAZ (Jasmonate ZIM-domain) repressor proteins bind to downstream bHLH transcription factors (predominantly MYC2, MYC3, and MYC4) together with co-repressor complexes composed of the NINJA adaptor protein, topless (TPL), and histone deacetylases (HDAs), blocking the transcription of JA-responsive defense genes [28,29]. When plants perceive stress signals (pathogen infection, herbivory, heavy metal toxicity, mechanical wounding), endogenous JA biosynthesis is rapidly activated through LOX-AOS-AOC-OPR serial enzymatic reactions to accumulate the JA-Ile ligand [16]. JA-Ile binds to the SCF-COI1 ubiquitin E3 ligase-JAZ co-receptor complex, triggering ubiquitination and 26S proteasome-mediated degradation of JAZ repressors. Subsequently, JA-Ile releases MYC transcription factors bound to G-box cis-elements on promoters of JA marker genes to activate downstream transcriptional cascades regulating antioxidant metabolism, osmolyte biosynthesis, metal detoxification, and secondary metabolite production [16,30].

JA signal transduction serves as a critical communication medium between host plants and beneficial AMF Symbiosis during root colonization and stress adaptation. Root JA concentration determines AMF colonization efficiency and arbuscule formation capacity. JA-deficient tomato mutants show drastically reduced AMF infection rate, while appropriate JA supplementation promotes AMF structure development [31]. In poplar–ectomycorrhizal symbiosis, overactivated *PtMYC2* inhibits mycorrhizal compatibility by repressing cell-wall remodeling and nutrient transporter genes, indicating tight JA signal dosage-dependent control over mutualistic symbiosis establishment [32]. Research on apple seedlings under cadmium stress reveals that AMF inoculation significantly upregulates multiple JA biosynthetic and signal transduction genes, improving JA-mediated antioxidant and metal detoxification pathways [31]. Nevertheless, critical knowledge gaps persist regarding how AMF symbiosis rewrites the genome-wide JA transcriptional landscape in woody poplars exposed to As stress, and which core transcription factors and downstream JA effector genes function as pivotal molecular switches linking AMF symbiotic signals and As tolerance in *P. tomentosa*.

To explore the molecular mechanism by which AMF symbiosis enhances the As tolerance of *P. tomentosa* through regulating JA biosynthesis and metabolism, we proposed the core scientific hypothesis that *R. irregularis* inoculation triggered genome-wide transcriptional reprogramming of key genes in the JA signaling pathway of *P. tomentosa* under As stress. A potted plant experiment with two factors (with or without AMF inoculation and As stress) was established. Comparative transcriptome sequencing of *P. tomentosa* seedlings from four experimental treatments identified differentially expressed genes (DEGs) responsive to As stress and AMF symbiosis, screened all JA biosynthesis and metabolism genes, and analyzed their expression dynamic patterns across treatments via heatmap visualization. Our results will elaborate the core correlation mechanism among AMF symbiosis, JA signal transduction, and As stress tolerance in woody phytoremediation species. The transcriptional data generated in this research will also provide technical parameters for field application of AMF–poplar combined remediation systems in As-polluted areas, facilitating the large-scale promotion of forest-based phytoremediation technology.

## 2. Materials and Methods

### 2.1. Biological Material and Growth Conditions

The experimental soil was collected from the 0–20 cm plow layer of the Botanical Garden at Henan University of Science and Technology (yellow fluvo-aquic soil). After air-drying, the soil was passed through a 2 mm sieve and autoclaved at 121 °C for 30 min; its initial pH was 7.0 ± 0.1. Sand was obtained from the river beach of Luopu Park, Luoyang. It was repeatedly washed with deionized water, air-dried, sieved to obtain 0.5–1.0 mm fractions, and autoclaved at 121 °C for 30 min prior to use. The organic component was a commercial seedling substrate (mainly composed of peat, perlite, and decomposed organic materials), purchased from a local agricultural supply market in Luoyang, Henan, with organic matter content ≥45%. The cultivation substrate was prepared by thoroughly mixing soil, sand, and organic substrate at a mass ratio of 1:1:1. The initial physicochemical properties of the cultivation substrate were determined using standard agricultural analytical methods. Briefly, the substrate pH was measured via potentiometry at a soil–water ratio of 2.5:1 (NY/T 1377-2007) [33]. The organic matter content was determined by the potassium dichromate external heating method (NY/T 1121.6-2006) [34]. Alkali-hydrolyzable nitrogen was measured using the alkali-diffusion method (LY/T 1228-2015) [35]. Available phosphorus was determined by sodium bicarbonate extraction combined with the molybdenum-antimony-ascorbic acid colorimetric method (NY/T 1121.7-2014) [36]. Available potassium was determined by ammonium acetate extraction and flame photometry (NY/T 889-2004 [37]). All measurements were performed in triplicate. The measured physicochemical indices of the mixed substrate were as follows: organic matter 50.52 g/kg, available potassium 83.35 mg/kg, alkali-hydrolyzable nitrogen 40.52 mg/kg, available phosphorus 8.17 mg/kg, and pH 7.8.

One-year-old fast-growing *Populus tomentosa* cuttings were collected from the Botanical Garden of Henan University of Science and Technology (Luoyang, China) and were authenticated as *P. tomentosa* by Associate Professor Qiaoming Zhang from the College of Horticulture and Plant Protection, Henan University of Science and Technology. Prior to transplanting, cuttings approximately 15 cm in length were surface-sterilized with 70% (*v*/*v*) ethanol for 15 s and subsequently rinsed five times with distilled water. The cutting seedlings were pre-germinated in moist, sterilized sand under a constant temperature of 25 °C over a 10-day incubation period. Uniform and healthy seedlings were screened and transplanted into plastic pots (25 cm diameter, 35 cm height). Each pot was filled with 5 kg of the prepared soil mixture. Cultivation was conducted in a greenhouse (April–June 2024) at 22–34 °C with a relative humidity of 70–85%. A weekly application of 100 mL of Hoagland’s solution (2.0 mmol/L) was provided to each pot, and soil water content was maintained at 75% field capacity by regular weighing and irrigation with deionized water.

The arbuscular mycorrhizal fungus (AMF) strain, *Rhizophagus irregularis* (Schenck and Smith, strain BGC BJ09), was obtained from the Beijing Academy of Agriculture and Forestry Sciences, Beijing, China. The mycorrhizal inoculum consisted of sandy soil, spores (approximately 53 g per kilogram of dry soil), extraradical hyphae, and colonized root fragments. For AMF-inoculated pots, 30 g of the *R. irregularis* inoculum was placed 3 cm beneath the *P. tomentosa* cutting seedling. For the non-AMF control treatment, each pot received 50 mL of microbial filtrate to normalize background microbial communities excluding viable AMF propagules. This filtrate was obtained by filtering a suspension of 30 g non-sterilized *R. irregularis* inoculum through a 10 μm filter membrane.

### 2.2. Experimental Design and Statistical Analysis

A two-factor completely randomized block design was used in this study. The two factors were AMF inoculation (non-inoculated control vs. *R. irregularis* inoculation) and arsenic stress (non-As control vs. 100 mg·kg^−1^ As, calculated based on dry substrate weight). Arsenic was supplied as Na_3_AsO_4_·12H_2_O solution, which was sprayed onto the substrate and thoroughly mixed to achieve homogeneous As distribution. Four treatments were established: (1) non-inoculated *P. tomentosa* under non-As stress (CK0); (2) non-inoculated *P. tomentosa* under As stress (CK100); (3) *R. irregularis*-inoculated *P. tomentosa* under non-As stress (Ri0); and (4) *R. irregularis*-inoculated *P. tomentosa* under As stress (Ri100). Each of the four treatments was replicated three times biologically, with one seedling per pot. Three replicates were arranged for each treatment, yielding a total of 12 pots. SPSS 16.0 (SPSS Inc., Chicago, IL, USA) was employed to conduct two-way ANOVA for statistical analysis. Results are shown as mean ± SD (n = 3). The overall experimental workflow, including plant cultivation, sampling, phenotypic measurement, and transcriptomic analysis, is illustrated in Figure S1.

### 2.3. AMF Colonization Assessment

AMF colonization was assessed using the lactic acid–glycerol trypan blue staining method [32,38]. Harvested root tissues were thoroughly rinsed with tap water to eliminate adhering soil particles and preserved in formalin–acetic acid–alcohol (FAA) fixative. Following 24 h of fixation, excess fixative was removed by washing with distilled water. Root fragments with appropriate thickness were selected and trimmed to around 1 cm in length. These root sections were cleared in 10% (*w*/*v*) KOH solution within a water bath maintained at 90 °C until tissues became transparent (roughly 1 h). After rinsing with distilled water, the samples were acidified in 1% (*v*/*v*) HCl for 5 min. Once the hydrochloric acid solution was decanted, root segments were stained with trypan blue at 90 °C for a 30 min incubation, followed by destaining in a lactic acid–glycerol mixture (1:1, *v*/*v*). The AMF colonization rate was quantified using the weighted root segment method. Briefly, 200 root segments were randomly subsampled from each treatment. Microscopic observation was performed to detect intraradical hyphae and vesicles, and the quantity of colonized root fragments was documented.

### 2.4. Plant Measurement and Root Morphology

Cutting seedlings of *P. tomentosa* were harvested after a three-month cultivation period following transplantation. Whole plants were thoroughly washed with deionized water and gently blotted dry, and plant height was measured with a graduated scale. Each seedling was divided into aboveground parts (stems and leaves) and roots, and fresh biomass of each fraction was weighed using an electronic balance. All tissues were then oven-dried at 70 °C until constant mass was achieved, after which shoot and root dry weights were documented. The root-to-shoot ratio was calculated as the quotient of root dry weight and shoot dry weight. Leaf area was determined by the coordinate paper method [39].

Root morphological traits of *P. tomentosa* were measured via digital scanning with an Epson Expression 12000 XL root scanner (Seiko Epson Co., Ltd., Tokyo, Japan). The obtained scanning data were further analyzed using the Win-RHIZO Pro 2017a root analysis software (Regent Instruments, Sainte Foy, QC, Canada). For measurements, *P. tomentosa* root samples from each treatment were carefully washed with tap water to eliminate attached soil particles, and special attention was paid to prevent breakage of fine roots. Afterwards, root systems were evenly spread out to minimize overlapping roots during scanning.

### 2.5. Total RNA Extraction, Library Preparation, and Illumina Sequencing

The whole seedlings were immersed in liquid nitrogen and ground in mortars. Total RNA was extracted from *P. tomentosa* root samples using the TRIzol^®^ Reagent (Invitrogen, Carlsbad, CA, USA) according to the manufacturer’s protocol. The integrity and potential DNA contamination of the extracted RNA were assessed by 1% agarose gel electrophoresis, with the brightness ratio of 28S rRNA to 18S rRNA being approximately 2.0, indicating high integrity. Purity and concentration were measured with a NanoDrop spectrophotometer ND-2000 (Thermo Fisher Scientific Inc., Waltham, MA, USA), with A260/A280 ratios around 2.0 and A260/A230 ratios between 1.8 and 2.0, confirming the absence of polysaccharide and protein contamination. Only high-quality RNA samples were used to construct the sequencing library. RNA-seq transcriptome libraries were prepared using the TruSeq RNA Sample Preparation Kit (Illumina Inc., San Diego, CA, USA) according to the manufacturer’s instructions, and RNA-Seq analysis was conducted using the Illumina NovaSeq 6000 platform at Majorbio Bio-pharm Technology Co., Ltd. (Shanghai, China), generating 150 bp paired-end reads. A total of 12 cDNA libraries were constructed and sequenced (four treatments—CK0, CK100, Ri0, and Ri100—each with three biological replicates). Each biological replicate corresponds to one independent individual plant. The same three independent plant samples per treatment were used as biological replicates for physiological measurements, RNA-seq library construction and subsequent bioinformatic analyses. Raw sequencing reads have been deposited in the Sequence Read Archive (SRA) of NCBI under BioProject accession PRJNA1252924.

### 2.6. Read Alignment, Functional Annotation, and Identification of Differentially Expressed Genes

Prior to read alignment, Cutadapt v2.10 software was used to cut off adapter sequences [8]. To obtain more reliable clean reads, reads containing N (undetermined bases), low-quality reads (Q-value ≤ 20), and poly-N were discarded. After filtering, FastQC v0.11.9 software was used to calculate basic quality metrics of the clean reads, including Q20, Q30, and GC content. Since *Populus tomentosa* lacks a high-quality annotated reference genome, we used the closely related *Populus trichocarpa* genome for read mapping, a common strategy for poplar transcriptome analysis. Given that a reference genome is available for poplar, HISAT2 v2.0.5 was employed to build the reference genome index and to align paired-end clean reads to the *Populus trichocarpa* reference genome (*P. trichocarpa*_v4.1) (https://www.ncbi.nlm.nih.gov/datasets/genome/GCF_000002775.5/ (accessed on 23 May 2026)), and the resulting SAM files were converted into BAM files for subsequent quantification. All expressed genes and transcripts were annotated through BLAST+ v2.9.0 against the databases NCBI protein nonredundant (NR), Swiss-Prot, Clusters of Orthologous Groups of proteins (COG), Gene Ontology (GO), and Kyoto Encyclopedia of Genes and Genomes (KEGG) at an E-value cut-off of 1 × 10^−5^.

To detect differentially expressed genes (DEGs) between different samples, the expression levels of each transcript were measured by the method of fragments per kilobase of exon per million mapped reads (FPKM). DESeq2 was used for gene differential expression analysis. To fully determine which genes were specifically modulated during AMF inoculation and/or As stress, the transcripts with adjusted *p*-value < 0.05 and |log_2_FoldChange| ≥ 1 were identified as DEGs between any of the groups.

### 2.7. Functional Enrichment of DEGs and Identification of Transcription Factors

To discern which functional categories of DEGs in *P. tomentosa* seedlings were regulated by As stress and *R. irregularis* inoculation, GO and KEGG enrichment analyses of DEGs were carried out. GO and KEGG functional enrichment analyses were conducted to identify significantly enriched DEGs in GO terms and KEGG metabolic pathways at *p*-value ≤ 0.05 under the whole-transcriptome background by Goatools (https://pypi.org/project/goatools/, 23 May 2026) and KOBAS (http://kobas.cbi.pku.edu.cn/home.do, 23 May 2026). TFs were identified with the Plant Transcription Factor Database v5.0 (http://planttfdb.cbi.pku.edu.cn/, 23 May 2026).

### 2.8. Co-Expression Network Analysis

Gene co-expression network analysis was performed for transcription factors (TFs) responsive to As stress and AMF inoculation in *P. tomentosa*. A set of 50 key TFs was screened based on the transcriptome sequencing data, and their expression values were normalized by log_2_(FPKM + 1) transformation. Expression clustering patterns across different treatments were visualized using TBtools v2.458. Gene pairs with a PCC *p*-value < 0.05 were collected and visualized to construct co-expression networks using Cytoscape 3.10.1 software. The topological overlap matrix (TOM) was generated based on the weighted gene co-expression network analysis (WGCNA) algorithm with a soft threshold power of 8. Pearson correlation coefficients (PCC) were derived from the WGCNA output. Significantly co-expressed pairs were screened with the criteria of TOM > 0.05 and |PCC| > 0.2, and core hub genes were identified by analyzing the network topological characteristics. Only TFs with a sub-network consisting of at least two genes were considered for further analysis.

## 3. Results

### 3.1. Mycorrhizal Colonization Rate

**Figure 1:**
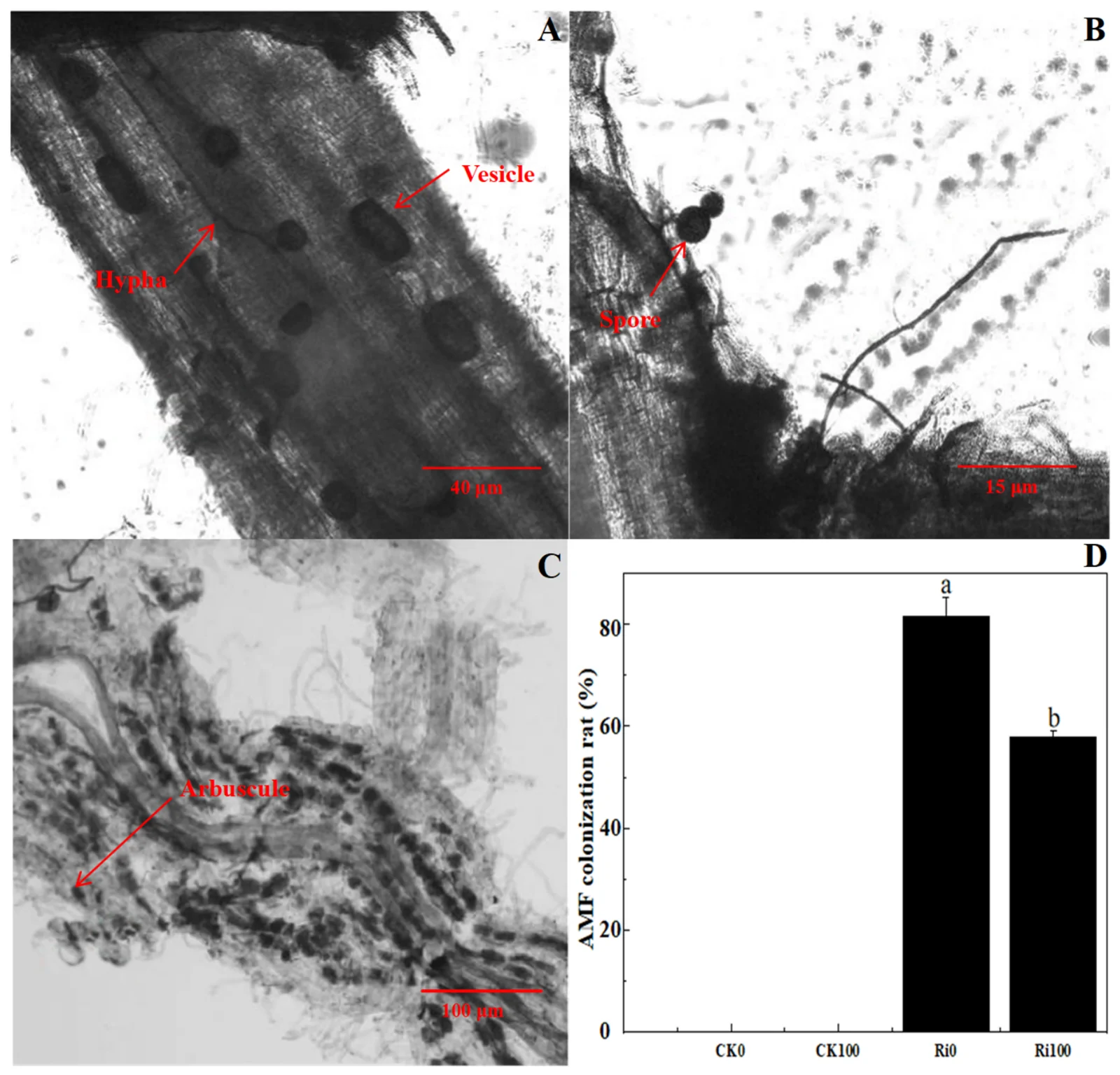
AMF morphology and colonization rate in *Populus tomentosa* roots under As stress. CK0: non-inoculation under non-As stress; CK100: non-inoculation under As stress; Ri0: *R. irregularis* inoculation under non-As stress; Ri100: *R. irregularis* inoculation under As stress. (**A**) Root segments showing hyphae and vessels (scale bar = 40 μm); (**B**) Hyphae and spores on the root surface (scale bar = 20 μm); (**C**) Arbuscules in root cortical cells (scale bar = 100 μm); (**D**) AMF colonization rate (%) under different treatments. Different lowercase letters above the bars indicate significant differences among treatments at *p* < 0.05 based on Duncan’s multiple range test.

Microscopic examination revealed that no AMF colonization was observed in any *P. tomentosa* roots from the non-inoculated treatments (CK0 and CK100). In contrast, following inoculation with *R. irregularis*, AMF established a distinct symbiotic relationship with *P. tomentosa* roots; AMF hyphae, vesicles, and spores were visible in *P. tomentosa* roots (Figure 1A,B), and arbuscules were present within the root cortical cells (Figure 1C). As stress inhibited *R. irregularis* colonization, the colonization rate in the *R. irregularis*-inoculated seedlings under As stress conditions (Ri100) decreased by 24% compared with the non-As stress treatment (Ri0) (Figure 1D).

### 3.2. Growth Parameters and Root Morphology

**Figure 2:**
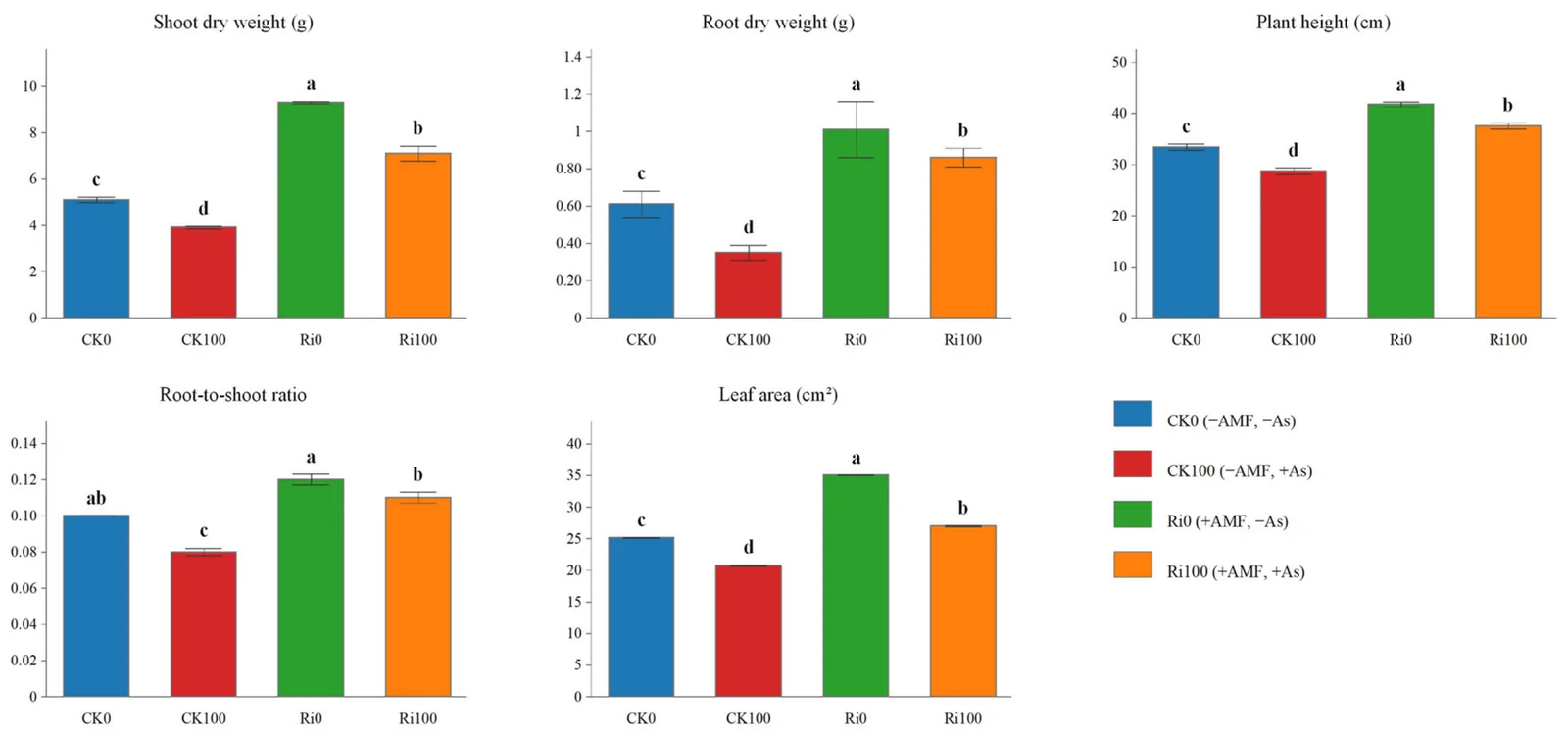
Effects of As stress and *Rhizophagus irregularis* inoculation on the growth parameters of *Populus tomentosa*. Different letters indicate significant differences (*p* < 0.05) using Tukey’s test; data are presented as mean ± SD (n = 3). CK0: non-inoculation under non-As stress; CK100: non-inoculation under As stress; Ri0: *R. irregularis* inoculation under non-As stress; Ri100: *R. irregularis* inoculation under As stress.

*R. irregularis* inoculation mitigated the adverse effects of As stress on the growth of *P. tomentosa* seedlings. As stress markedly inhibited the growth of *P. tomentosa* seedlings (Figure 2). For non-inoculated plants, As exposure caused substantial declines in plant height (14.19%), root-to-shoot ratio (20.00%), shoot dry weight (23.53%), root dry weight (42.62%), and leaf area (18.00%) in CK100 relative to CK0. By contrast, in *R. irregularis*-inoculated seedlings, the inhibitory effects of As stress were mitigated; plant height, root-to-shoot ratio, shoot dry weight, and root dry weight decreased by only 10.13%, 8.33%, 23.66%, and 14.85%, respectively, when comparing Ri100 with Ri0, whereas *R. irregularis* inoculation markedly increased plant height, root-to-shoot ratio, and total dry weight, thereby alleviating the As-induced inhibition (Figure 2). Under As stress, *R. irregularis*-inoculated seedlings (Ri100) showed increases in plant height (by 30.79%), root-to-shoot ratio (by 37.5%), shoot dry weight (by 82.05%), and root dry weight (by 145.71%) compared with the non-inoculated control (CK100). Even in the absence of As stress, *R. irregularis* inoculation (Ri0) still promoted seedling growth. Compared with CK0, Ri0 showed increases in plant height (by 24.94%), root-to-shoot ratio (by 20.00%), shoot dry weight (by 82.35%), and root dry weight (by 65.57%) relative to the non-inoculated control (CK0).

*R. Irregularis* inoculation mitigated the adverse effects of As stress on root morphology of *P. tomentosa* seedlings (Figure 2). Arsenic stress exerted strong suppressive effects on root development. For inoculated seedlings, As exposure still triggered obvious root-growth inhibition. When comparing Ri100 with Ri0, As stress reduced total root length (by 49.26%), root surface area (by 21.68%), root volume (by 48.70%), root diameter (by 16.67%), root tips (by 88.22%), forks (by 46.04%) and crossings (by 69.48%). Nevertheless, *R. irregularis* inoculation greatly counteracted As-provoked root-growth suppression under arsenic stress. In direct comparison under As stress, *R. irregularis*-inoculated seedlings exhibited increases in total root length (by 11.07%), root surface area (by 23.08%), root volume (by 26.40%), root tips (by 31.73%), forks (by 25.66%) and crossings (by 49.71%), compared with the non-inoculated As stress control (CK100). Even without As stress, *R. irregularis* inoculation exerted positive effects on root architecture. Compared with CK0, Ri0 displayed increases in total root length (by 27.10%), root surface area (by 21.19%), root volume (by 24.19%), root diameter (by 6.67%), root tips (by 23.02%), forks (by 35.04%) and crossings (by 77.98%) relative to the non-inoculated control (CK0). Collectively, these results indicate that *R. irregularis* inoculation improves root system performance both under normal growth and As-stress conditions and relieves As-induced root damage (Figure 2).

**Figure 3:**
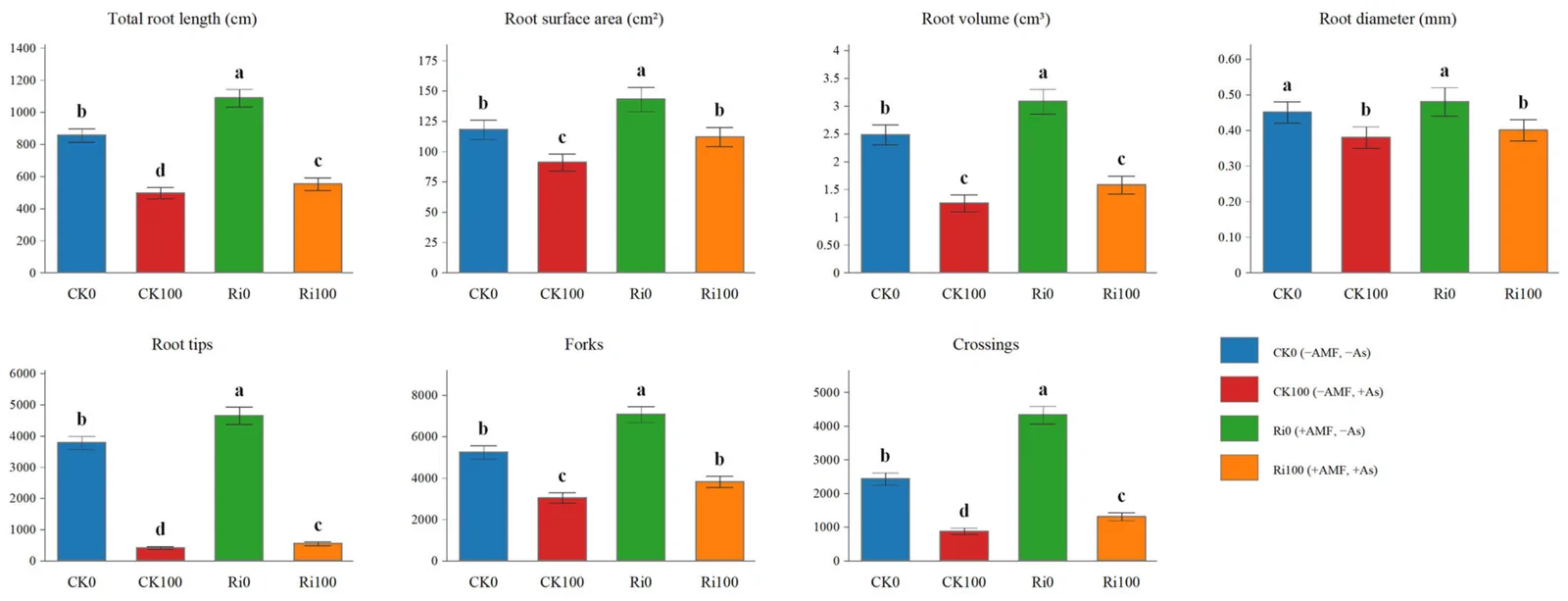
Effects of As stress and *Rhizophagus irregularis* inoculation on morphological parameters of *Populus tomentosa* roots. Different letters indicate significant differences (*p* < 0.05) using Tukey’s test; data are presented as mean ± SD (n = 3). CK0: non-inoculation under non-As stress; CK100: non-inoculationunder As stress; Ri0: *R. irregularis* inoculation under non-As stress; Ri100: *R. irregularis* inoculation under As stress.

Two-way ANOVA was performed to evaluate the main and interactive effects of As stress and *R. irregularis* inoculation on mycorrhizal colonization rate and growth-related parameters of *P. tomentosa* (Figure 3). Both As stress and AMF inoculation had highly significant main effects (*p* < 0.01) on mycorrhizal infection rate, root dry weight, plant height, root length, root area, root volume, and the number of root tips. For total root absorption area, root diameter, number of forks, and number of crossings, As stress produced highly significant main effects (*p* < 0.01), while AMF inoculation had significant main effects at *p* < 0.05. A significant interaction between As and AMF inoculation (*p* < 0.01) was detected for mycorrhizal infection rate and root area. A significant AMF × As interaction (*p* < 0.05) was also observed for root diameter. By contrast, no significant interactive effects (NS) of As and AMF were found for root dry weight, plant height, total root absorption area, root length, root volume, number of root tips, number of forks and number of crossings.

### 3.3. Transcriptome Sequencing and Gene Annotation

RNA-seq was used to investigate the transcriptomic responses of *P. tomentosa* seedlings inoculated with *R. irregularis* under As stress. Twelve cDNA libraries covering four treatments (CK0, CK100, Ri0, and Ri100) with three biological replicates each were sequenced (Table S1). After filtering low-quality reads and removing adapters, all samples displayed robust sequencing quality: clean read ratios reached 98.74–98.77%, Q20 ranged from 98.38 to 98.53%, Q30 exceeded 94.93%, and GC content varied from 43.54% to 44.09%. Clean reads were aligned to the *P. tomentosa* reference genome, with overall mapping rates of 79.12–82.76%. Uniquely mapped reads accounted for 76.90–80.18% of clean reads, while multi-mapped reads only made up 2.07–3.01%, confirming reliable mapping performance (Table S1).

Homology searches against GO, KEGG, COG, NR, Swiss-Prot, and Pfam databases were conducted to annotate expressed genes and transcripts (Table S2). We identified 30,612 expressed genes and 51,955 expressed transcripts, and 99.14% of expressed genes and 99.45% of expressed transcripts received functional annotations. For the full transcriptome set, 97.92% of all genes and 98.73% of all transcripts were annotated across the six databases. NR provided the highest annotation coverage for both expressed genes (99.14%) and transcripts (99.44%), followed by COG (94.70% and 95.40%). KEGG yielded the lowest matching ratio (48.36% for genes, 48.95% for transcripts). GO, Swiss-Prot, and Pfam delivered moderate annotation rates, laying a foundation for subsequent DEG functional enrichment analysis.

### 3.4. Analysis of Differentially Expressed Genes Associated with R. irregularis Inoculation and As Stress

**Figure 4:**
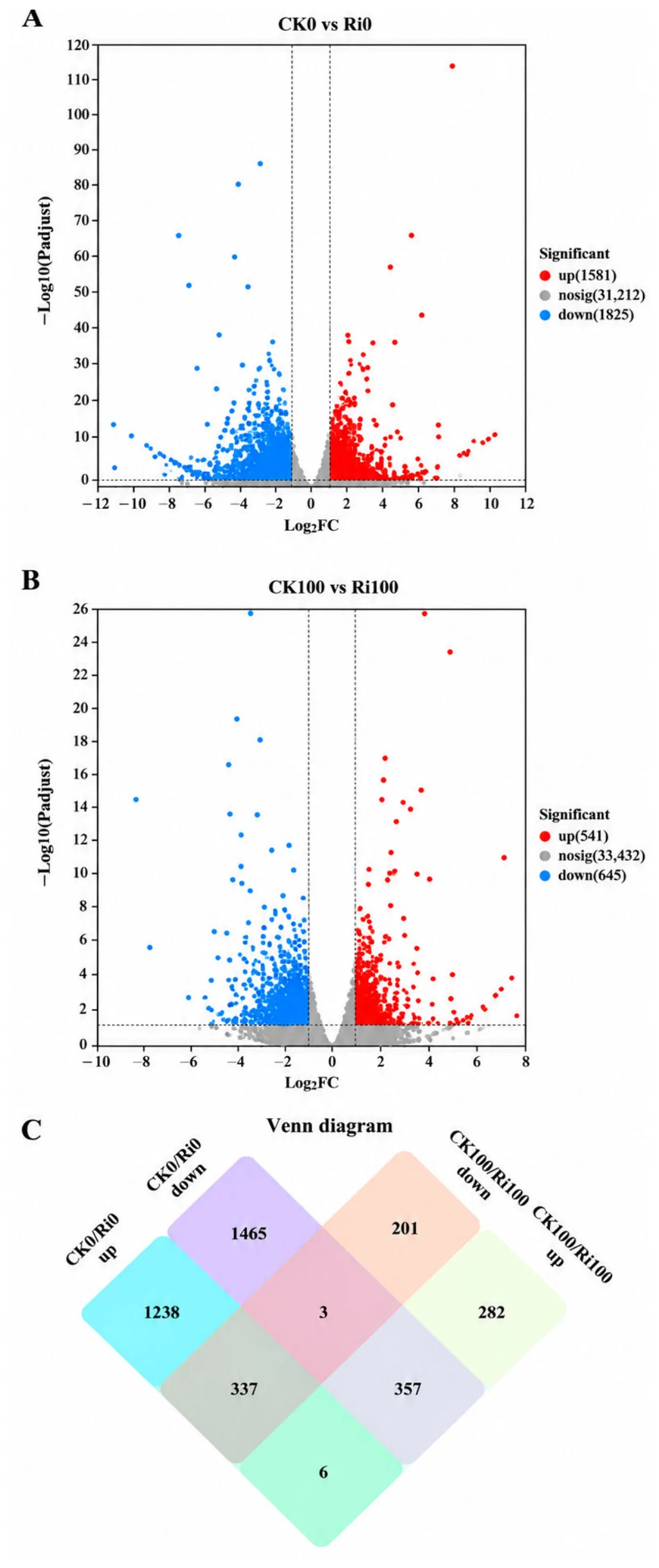
Analysis of upregulated and downregulated DEGs of *Populus tomentosa* induced by *Rhizophagus irregularis* inoculation and As stress. (**A**) Volcano plot of DEGs in the CK0 vs. Ri0 comparison. (**B**) Volcano plot of DEGs in the CK100 vs. Ri100 comparison. Red dots represent significantly upregulated genes, blue dots represent significantly downregulated genes, and gray dots represent non-significantly expressed genes. (**C**) Venn diagram illustrating the overlaps among up- and downregulated DEGs in the two comparisons. CK0: non-inoculation under non-As stress; CK100: non-inoculationunder As stress; Ri0: *R. irregularis* inoculation under non-As stress; Ri100: *R. irregularis* inoculation under As stress.

Pairwise comparisons of gene expression profiles across different treatments were conducted to screen differentially expressed genes (DEGs) responding to *R. irregularis* inoculation and As stress. These comparative analyses provided novel insights into the symbiotic interactions between AMF and *P. tomentosa* subjected to As stress (Figure 4). Without As stress, *R. irregularis* inoculation induced 3406 DEGs, consisting of 1581 upregulated and 1825 downregulated genes (Figure 4A). When exposed to As stress, *R. irregularis* inoculation generated 1186 DEGs, among which 541 genes were upregulated and 645 were downregulated (Figure 4B).

A Venn diagram was constructed to further explore the relationships among the 3889 DEGs derived from four distinct comparisons (Figure 4C). The comparison between CK100 and Ri100 groups identified 645 upregulated DEGs (282, 357, and 6) and 541 downregulated DEGs (201, 3, and 337). Furthermore, the 3406 DEGs (1581 upregulated and 1825 downregulated) detected from the CK0 vs. Ri0 comparison exhibited distinct profiles relative to those obtained from the CK100 versus Ri100 group. Among *R. irregularis*-inoculated seedlings under non-As-stressed conditions, nine DEGs (six upregulated and three downregulated) showed expression patterns unaffected by As stress. Within the CK100 versus Ri100 comparison, 483 DEGs (282 upregulated and 201 downregulated) displayed consistent expression trends compared with the DEGs identified in the CK0 vs. Ri0 group. Additionally, 694 DEGs (337 downregulated and 357 upregulated) showed reversed expression patterns between As and non-As stress treatments. Collectively, 1177 DEGs, consisting of 483 As stress-specific transcripts and 694 trend-reversed genes, were considered key participants mediating the responses of *R. irregularis*-inoculated seedlings to As stress.

### 3.5. Functional Annotation of DEGs Induced by R. irregularis Inoculation and As Stress

**Figure 5:**
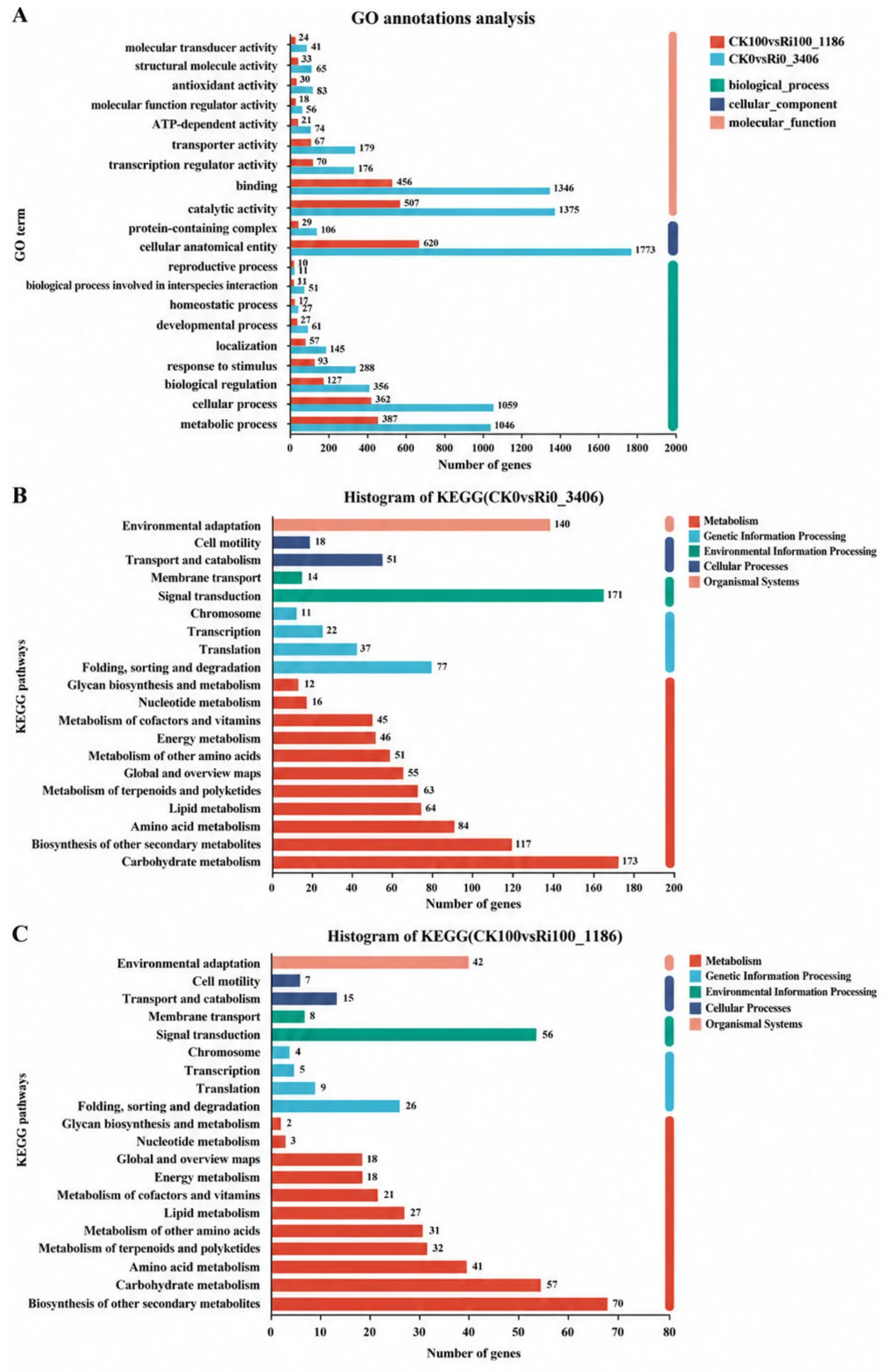
Functional annotation analysis of DEGs of *Populus tomentosa* induced by *Rhizophagus irregularis* inoculation under As stress (**A**) Significantly enriched biological process, molecular function and cellular component GO terms of DEGs in CK100 vs. Ri100 and CK0 vs. Ri0 comparison. (**B**) The histogram of KEGG in the CK0 vs. Ri0 comparison. (**C**) The histogram of KEGG in the CK100 vs. Ri100 comparison. CK0: non-inoculation under non-As stress; CK100: non-inoculationunder As stress; Ri0: *R. irregularis* inoculation under non-As stress; Ri100: *R. irregularis* inoculation under As stress.

To elucidate the mechanisms underlying the effect of *R. irregularis* inoculation on *P. tomentosa* seedlings under As stress, we selected the 1186 upregulated and downregulated DEGs from the CK100 versus Ri100 comparison for GO and KEGG annotation evaluation (Figure 5). Among these 1186 DEGs, 970 were successfully annotated to one or more Gene Ontology (GO) terms covering three major ontologies: biological process (BP), cellular component (CC), and molecular function (MF) (Figure 5A). Within the BP category, the most enriched functional terms included “cellular process” (362), “metabolic process” (387), “biological regulation” (127), and “response to stimulus” (93). For the CC category, the vast majority of DEGs were annotated to “cellular anatomical entity” (620), with a small subset corresponding to “protein-containing complex” (29). As for the MF category, “catalytic activity” (507), “binding” (456), and “transcription regulator activity” (70) constituted the top annotated terms.

KEGG pathway annotation indicated that 492 out of the 1186 DEGs were assigned to five primary pathway classes, including metabolism (M), environmental information processing (EIP), organismal systems (OS), cellular processes (CP), and genetic information processing (GIP) (Figure 5B). Within the GIP group, “folding, sorting and degradation” (26 genes) occupied the largest proportion in the GIP category. For the CP class, “transport and catabolism” (15 genes) was the primary subclass. “Environmental adaptation” (42 genes) represented the major subclass within OS, whereas “signal transduction” (56 genes) was predominant in EIP. As for the metabolism group, DEGs were predominantly enriched in “biosynthesis of other secondary metabolites” (70), “carbohydrate metabolism” (57), “amino acid metabolism” (41), “metabolism of terpenoids and polyketides” (32), “metabolism of other amino acids” (31), “lipid metabolism” (27), and “energy metabolism” (18).

In the CK0 versus Ri0 comparison, a comprehensive functional analysis was performed on 3406 DEGs using GO and KEGG annotations. In total, 2740 were successfully annotated to one or more GO terms, covering biological process (BP), cellular component (CC), and molecular function (MF) (Figure 5A). Within the BP ontology, the top enriched terms included “cellular process” (1059), “metabolic process” (1046), “biological regulation” (355), “response to stimulus” (288), and “localization” (145). For the CC ontology, “cellular anatomical entity” (1773) and “protein-containing complex” (106) constituted the two major subgroups. In the MF category, “binding” (1346), “catalytic activity” (1375), “transporter activity” (179), and “transcription regulator activity” (176) represented the predominant functional classifications.

Subsequent KEGG analysis showed that 1267 out of 3406 DEGs from the CK0 versus Ri0 comparison were allocated to five pathway classes: metabolism (M), environmental information processing (EIP), organismal systems (OS), cellular processes (CP), and genetic information processing (GIP) (Figure 5C). The metabolism category featured abundant DEGs associated with “carbohydrate metabolism” (173) and “lipid metabolism” (64). “Environmental adaptation” (140) was the leading subclass in the OS group, “transport and catabolism” (51) dominated the CP group, and “signal transduction” (171) was the most prominent subclass within EIP. In terms of GIP, “folding, sorting and degradation” (77), “translation” (37), and “transcription” (22) were the most highly annotated subcategories.

### 3.6. Functional Enrichment of DEGs Induced by R. irregularis Inoculation and As Stress

**Figure 6:**
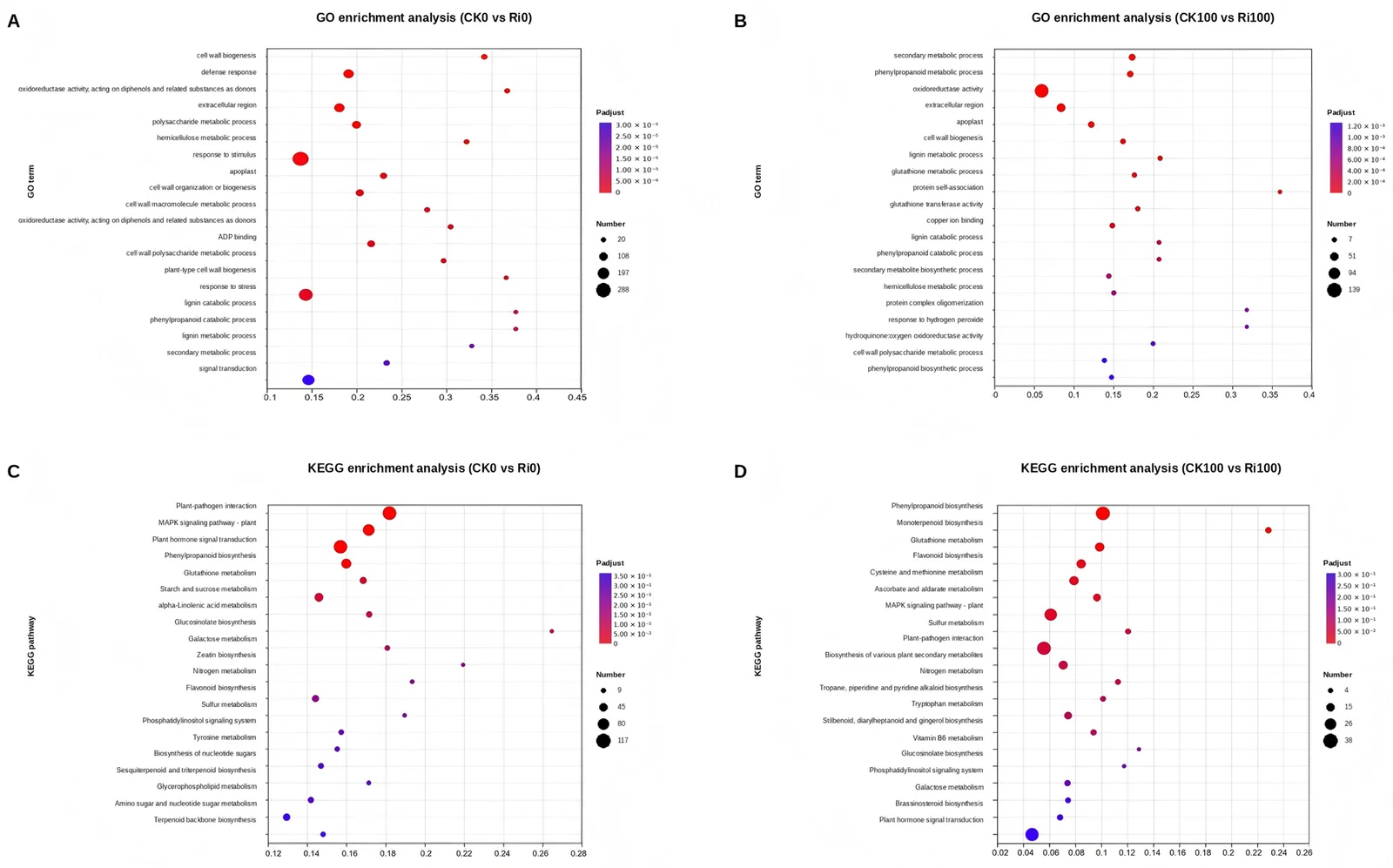
Functional enrichment analysis of DEGs of *Populus tomentosa* induced by *Rhizophagus irregularis* inoculation and As stress. (**A**) The GO enrichment bubble plot of DEGs in CK0 vs. Ri0. (**B**) The GO enrichment bubble plot of DEGs in CK100 vs. Ri100. (**C**) The KEGG pathway enrichment bubble plot of DEGs in CK0 vs. Ri0. (**D**) The KEGG enrichment bubble plot of DEGs in CK100 vs. Ri100. CK0: non-inoculation under non-As stress; CK100: non-inoculationunder As stress; Ri0: *R. irregularis* inoculation under non-As stress; Ri100: *R. irregularis* inoculation under As stress.

GO enrichment analysis was performed to elucidate the functions of differentially expressed genes (DEGs), which were classified into three categories: biological process, cellular component, and molecular function (Figure 6). The figure displays the top 20 significantly enriched GO terms (*p* ≤ 0.05) among DEGs. In the CK0 vs. Ri0 comparison, the most significantly enriched terms were oxidoreductase activity (19), oxygen as acceptor (11), and xyloglucan: xyloglucosyl transferase activity (16), while the terms with the largest gene counts were lignin catabolic process (20) and phenylpropanoid catabolic process (20) (Figure 6A). In contrast, in the CK100 vs. Ri100 comparison, the most significantly enriched terms were lignin metabolic process (14), phenylpropanoid catabolic process (11), and lignin catabolic process (11), whereas the terms with the largest gene counts were glutathione metabolic process (15), glutathione transferase activity (15), secondary metabolic process (29), and phenylpropanoid metabolic process (25) (Figure 6B).

KEGG pathway enrichment analysis revealed that 1143 (CK0 vs. Ri0) and 612 (CK100 vs. Ri100) DEGs were uniquely enriched. The significantly enriched KEGG pathways (*p* ≤ 0.05) are presented in Figure 6, with a total of 14 pathways in the first category. For CK0 vs. Ri0, the first-category pathways involving the largest numbers of DEGs were plant hormone signal transduction (114), plant–pathogen interaction (117), and MAPK signaling pathway–plant (84) (Figure 6C). For CK100 vs. Ri100, the first-category pathways with the largest numbers of DEGs were phenylpropanoid biosynthesis (38), plant–pathogen interaction (36), and MAPK signaling pathway–plant (30) (Figure 6D).

### 3.7. Expression Patterns of DEGs in P. tomentosa Related to Jasmonic Acid Biosynthesis and Metabolism Regulated by R. irregularis Inoculation and As Stress

**Figure 7:**
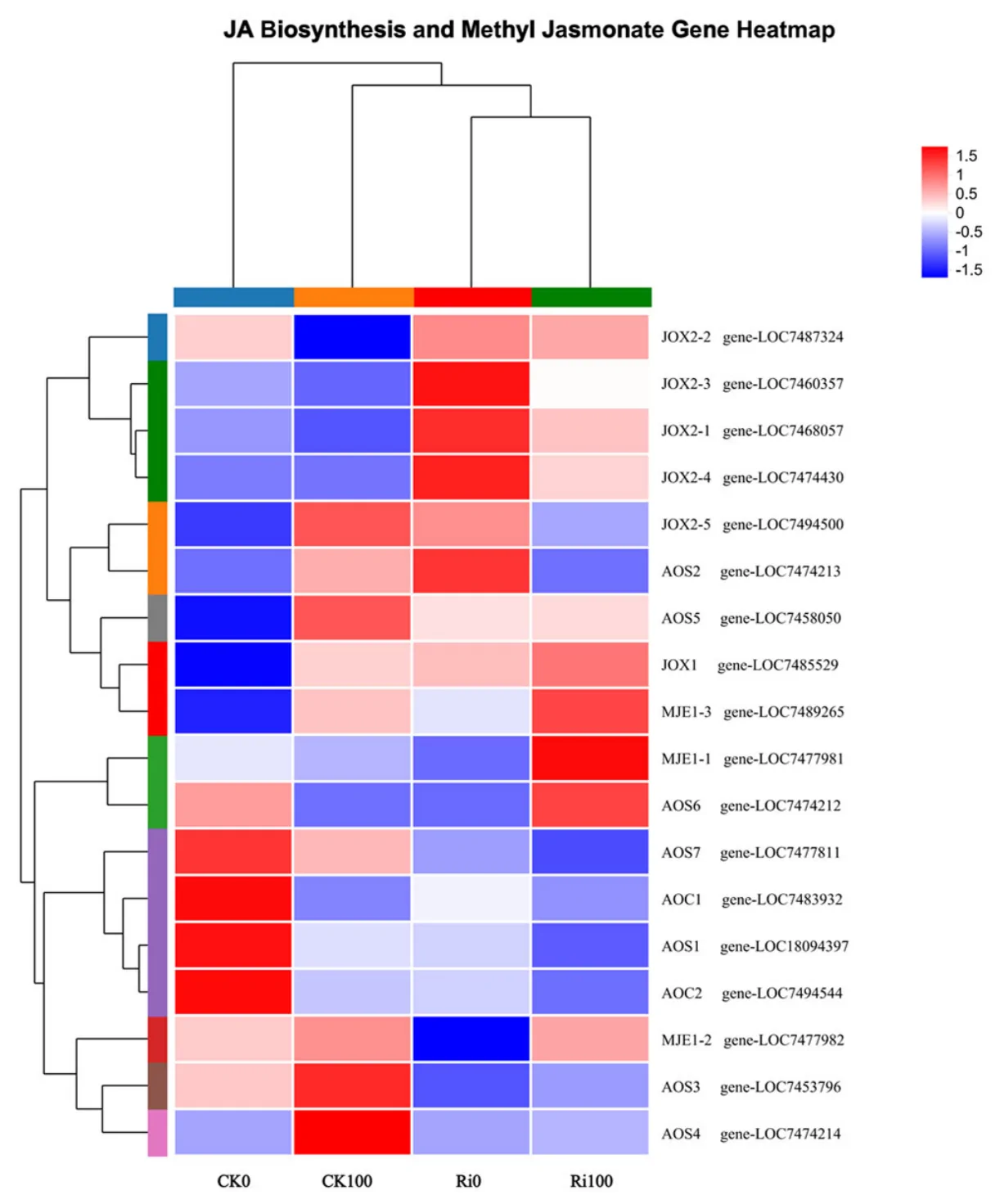
Heatmap of the expression patterns of DEGs in *Populus tomentosa* related to jasmonic acid biosynthesis and metabolism regulated by *Rhizophagus irregularis* inoculation and As stress. Each column corresponds to an individual sample, and each row denotes a single gene. The color gradient in the heatmap reflects the normalized gene expression level across different samples. CK0: non-inoculation under non-As stress; CK100: non-inoculationunder As stress; Ri0: *R. irregularis* inoculation under non-As stress; Ri100: *R. irregularis* inoculation under As stress. JOX: Jasmonate-induced oxygenase; AOS: Allene oxide synthase; AOC: Allene oxide cyclase; MJE: Methyl jasmonate esterase.

To further elucidate the molecular mechanisms underlying AMF-alleviated As detoxification in *P. tomentosa* seedlings, we characterized the expression profiles of DEGs involved in jasmonic acid (JA) biosynthesis and metabolism, including key enzyme-encoding genes from the JOX, AOS, and AOC gene families. Cluster analysis of these candidate genes identified 18 JA-pathway-related genes exhibiting distinct expression patterns among the four experimental treatments (Figure 7). Under non-As stress, *R. irregularis* inoculation exerted distinct regulatory effects on different subgroups of JA synthetic genes. *R. irregularis* inoculation markedly upregulated the transcription of *JOX2-1*, *JOX2-3*, *JOX2-4*, *AOS2*, and *AOS7*, while significantly suppressing the expression of *JOX2-2*, *JOX2-5*, *AOS1*, *AOC1*, *AOC2*, and *MJE1-2*. Meanwhile, the expression levels of *AOS3*, *AOS4*, *AOS5*, *AOS6*, *JOX1*, *MJE1-1*, and *MJE1-3* exhibited no obvious suppression or activation upon *R. irregularis* colonization under non-As stress.

Under As stress, *R. irregularis* inoculation also triggered widespread transcriptional reprogramming of JA synthetic genes. Inoculation with *R. irregularis* strongly induced the expression of *JOX2-1*, *JOX2-2*, *JOX2-3*, *JOX2-4*, *JOX1*, *AOS6*, and *MJE1-1*, whereas it repressed the expression of *JOX2-5*, *AOS2*, *AOS7*, *AOS1*, *AOC1*, *AOC2*, and *MJE1-2*. Genes including *AOS3*, *AOS4*, *AOS5*, and *MJE1-3* displayed moderate transcriptional changes following *R. irregularis* inoculation under As exposure.

When comparing As stress responses in non-inoculated *P. tomentosa* seedlings, As stress significantly downregulated *JOX2-1*, *JOX2-2*, *JOX2-3*, *JOX2-4*, *JOX2-5*, *AOS2*, *AOS5*, and *AOS6*, and activated the transcription of *JOX1*, *AOS1*, *AOC1*, *AOC2*, *AOS7*, *MJE1-2*, and *MJE1-3*. Genes *AOS3* and *AOS4* showed only slight expression fluctuations under As treatment without AMF. For *R. irregularis*-inoculated *P. tomentosa* seedlings, As stress induced the expression of *JOX2-1*, *JOX2-3*, *JOX2-4*, *AOS3*, *AOS4*, *AOS6*, and *MJE1-1*, and repressed the expression of *JOX2-2*, *JOX2-5*, *AOS2*, *AOS5*, *AOS7*, *JOX1*, *AOC1*, *AOC2*, *MJE1-2*, and *MJE1-3*.

### 3.8. Identification and Co-Expression Network Analysis of Transcription Factors (TFs) Associated with R. irregularis Inoculation and As Stress

**Figure 8:**
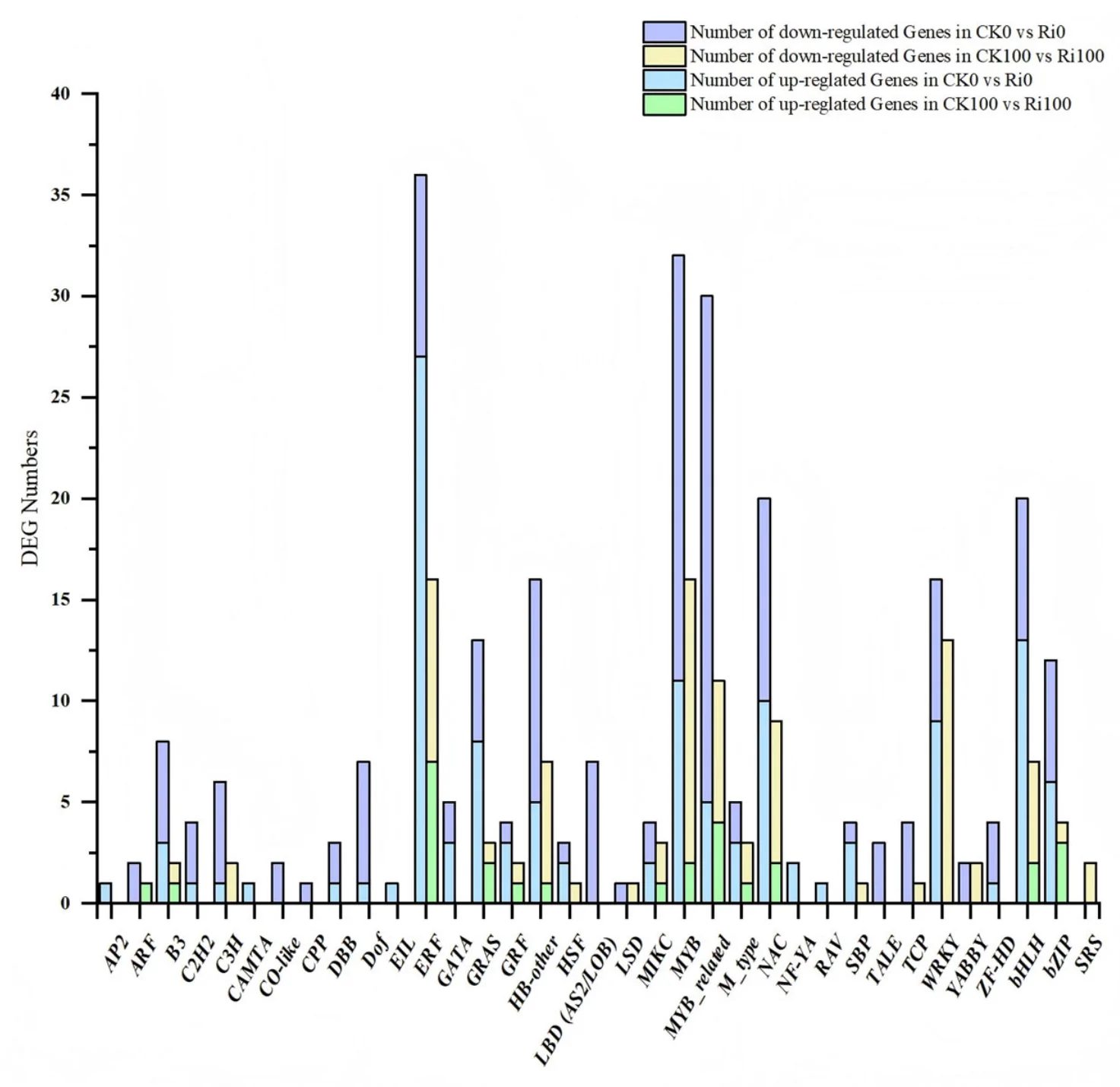
Analysis of DEG numbers of transcription factors (TFs) in CK100 vs. Ri100 and CK0 vs. Ri0 comparison. *X*-axis represents the TF family, and the *Y*-axis represents DEG numbers. CK0: non-inoculation under non-As stress; CK100: non-inoculationunder As stress; Ri0: *R. irregularis* inoculation under non-As stress; Ri100: *R. irregularis* inoculation under As stress.

To gain insight into the mechanisms by which *R. irregularis* inoculation alleviated As stress in *P. tomentosa* seedlings, differentially expressed genes encoding transcription factor proteins were identified. These TFs were assigned to multiple TF families, mainly including ERF, GATA, MYB, MYB-related, NAC, bZIP and WRKY (Figure 8). In the CK100 versus Ri100 comparison, *R. irregularis* inoculation induced widespread upregulation of DEGs within ERF, GATA, WRKY and NAC families, whereas several members of MYB-related and bZIP families were downregulated. Among them, the GATA family possessed the largest number of upregulated TFs. Similarly, in the CK0 versus Ri0 comparison, AMF inoculation triggered upregulation of abundant DEGs from ERF, GATA, MYB-related, and NAC families, while downregulated transcripts were predominantly distributed in MYB and bZIP families. The ERF family contained the maximum count of upregulated DE-TFs under non-As stress symbiotic conditions. Under As stress alone, multiple TF families, including ERF, MYB, HB, GRAS, NAC, bZIP, bHLH, and WRKY, were significantly involved in the As stress response in *P. tomentosa* seedlings. In the *R. irregularis*-inoculated groups, a greater number of differentially expressed TFs were annotated in the ERF, GRAS, MYB, HB, WRKY, bHLH, and bZIP families.

**Figure 9:**
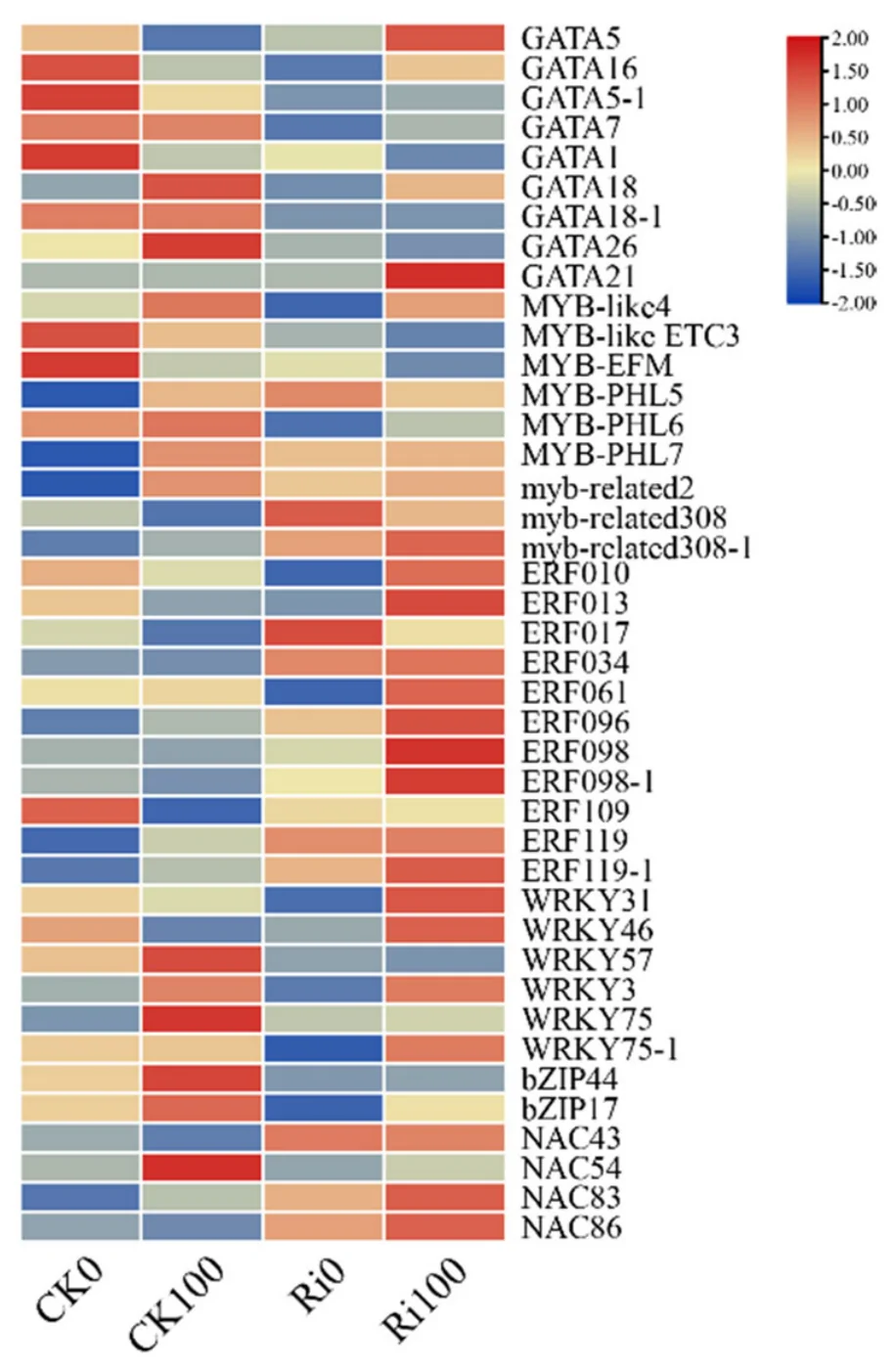
Heatmap of the gene expression patterns of transcription factors in *Populus tomentosa* regulated by *Rhizophagus irregularis* inoculation and As stress. Each column corresponds to an individual sample, and each row denotes a single gene. The color gradient in the heatmap reflects the normalized gene expression level across different samples. CK0: non-inoculation under non-As stress; CK100: non-inoculationunder As stress; Ri0: *R. irregularis* inoculation under non-As stress; Ri100: *R. irregularis* inoculation under As stress. GATA: GATA-binding; MYB: Myeloblastosis; MYB-EFM: Myeloblastosis-early flowering MYB protein; MYB-PHL: Myeloblastosis-phosphate starvation response-like; ERF: Ethylene-responsive; WRKY: W (Tryptophan)-R (Arginine)-K (Lysine)-Y (Tyrosine); bZIP: basic-leucine zipper; NAC: NAM-ATAF1-CUC2.

Based on the expression profiles of TFs from the CK0, CK100, Ri0, and Ri100 samples, a gene expression clustering heatmap was generated (Figure 9). A total of 41 differentially expressed TFs were screened and exhibited distinct expression patterns across the four experimental treatments (Figure 9). Under As stress, *R. irregularis* inoculation triggered obvious transcriptional reprogramming of these TFs. A large set of TFs, including multiple GATA, WRKY, and ERF family members, were markedly upregulated in the Ri100 group. Many of these induced TFs, such as *GATA5*, *WRKY31* and *ERF119*, were reported to participate in plant abiotic stress responses and symbiotic regulation. Meanwhile, several TFs belonging to the NAC and MYB families were significantly downregulated upon *R. irregularis* inoculation under As exposure. Notably, some GATA and bZIP members displayed strong induction specifically under non-stressed inoculated conditions (Ri0), indicating their potential roles in AM-symbiosis establishment independent of As stress.

**Figure 10:**
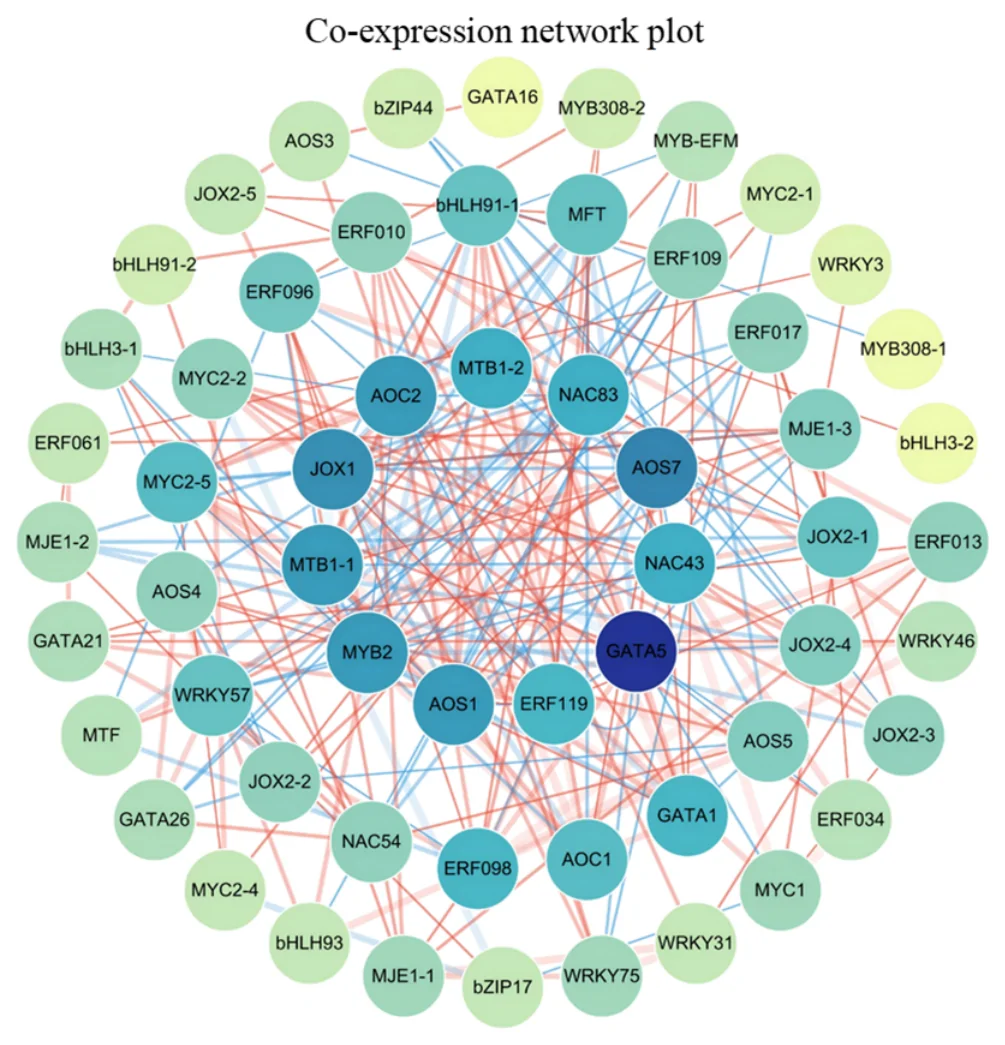
Co-expression network analyses of TFs and genes related to jasmonic acid biosynthesis and metabolism in *Populus tomentosa* regulated by *Rhizophagus irregularis* inoculation and As stress. Red and blue lines denote positive correlation and negative correlation, respectively.

Co-expression network analysis was performed to characterize the regulatory interactions between key TFs and significant jasmonic acid biosynthetic and metabolic genes using weighted gene co-expression network analysis (WGCNA). Only TFs linked to no fewer than two target genes in the sub-network were retained for subsequent analysis. The constructed co-expression network consisted of 26 JA biosynthetic and metabolic genes and 38 core TF nodes, forming 94 significant interaction edges in the final filtered sub-network (Figure 10). It should be noted that co-expression reflects association rather than direct regulatory interaction. ERF-family TFs exhibited the greatest number of connecting edges within this main co-expression network, followed by MYB, NAC, GATA, WRKY, and bZIP families, suggesting these TFs may modulate JA biosynthesis–metabolism upon *R. irregularis* inoculation and As stress. Both positive and negative co-expression correlations were detected between numerous transcription factors and JA-pathway genes, indicating dual regulatory modes of transcriptional activation and repression. Moreover, multiple functionally critical DEGs involved in JA biosynthesis and metabolism were identified to be co-expressed with distinct transcription factors. *AOC2* and *JOX1* were tightly co-expressed with *MTB1-1* and *MYB2*, whereas *AOS7* was correlated with *NAC83* and *NAC43*. In addition, *AOS1* and *AOC1* showed co-expression relationships with *ERF119* and *GATA1*.

## 4. Discussion

Arsenic (As) is a widely distributed toxic metallic element in the pedosphere that exerts a strong inhibitory effect on plant growth and development. However, arbuscular mycorrhizal fungi (AMF) enhanced the heavy metal tolerance of host plants through multiple pathways. Using *Populus tomentosa* seedlings as the experimental material, this study investigated the effects of inoculation with *Rhizophagus irregularis* under As stress on plant growth, root morphology, transcriptomic responses, and the associated transcriptional regulatory network. The results confirmed that *R. irregularis* could successfully establish mutualistic endosymbiotic associations with the roots of *P. tomentosa* and effectively alleviate As toxicity through synergistic regulation at both physiological and molecular levels, with the jasmonic acid (JA) biosynthetic and metabolic pathway and specific transcription factor networks playing pivotal roles in this process. Our results provided insights into how AMF modulated the molecular networks to enhance As tolerance in woody *P. tomentosa*, and expanded the understanding of AMF-mediated As detoxification mechanisms in deciduous tree species.

### 4.1. AMF Colonization Dynamics in P. tomentosa Roots Under As Stress

As stress had negative effects on AMF colonization capacity, which was a well-documented phenomenon in mycorrhizal symbiosis systems [40,41]. Microscopic observations in this study revealed no mycorrhizal structures in non-inoculated *P. tomentosa* roots, whereas typical AMF structures, including intraradical hyphae, vesicles, spores, and arbuscules, were clearly detected in *P. tomentosa* roots inoculated with *R. irregularis*. However, under As stress (Ri100), the mycorrhizal colonization rate decreased by 24% relative to the non-As-stressed group (Ri0), confirming that As stress suppressed mycorrhizal colonization in *P. tomentosa* roots (Figure 1). Two-way ANOVA statistical analysis further supported that As stress exerted highly significant main effects (*p* < 0.01) on mycorrhizal colonization rate, and a significant interaction existed between As stress and AMF inoculation (*p* < 0.01) (Table 1). This result was consistent with previous studies investigating host plants exposed to As contamination. For instance, elevated soil As concentrations decreased mycorrhizal colonization in Medicago sativa roots, an effect attributed to As-mediated inhibition of spore germination, hyphal proliferation, and arbuscule development [23]. Comparable observations have also been documented in safflower, where *R. irregularis* inoculation had higher root colonization than *Funneliformis mosseae* under As stress [42], as well as in cotton, in which *R. irregularis* maintained symbiosis accompanied by the reprogramming of Ca^2+^-signaling pathways upon As exposure [43]. As-metalloid toxicity damaged mycorrhizal cell membranes, interfered with energy metabolism, and disrupted signal exchange between host roots and symbiotic fungi, consequently lowering colonization rate. It also acted as a phosphate (Pi) analog, perturbed host Pi sensing, and imposed oxidative pressure that could inhibit AMF spread in the root cortex [44]. It was noted that even with a 24% reduction in colonization rate under As stress, *R. irregularis* still successfully established functional symbiosis with *P. tomentosa* roots and delivered stress-alleviation benefits, indicating that *R. irregularis* possessed moderate intrinsic As tolerance, making it a promising inoculum candidate for phytoremediation application in As-polluted sites [45].

### 4.2. The Alleviating Effects of R. irregularis and As Stress on the Growth Parameters and Root Morphology of P. tomentosa

In this study, growth parameter and root morphology measurements clearly illustrated that As stress severely impaired seedling performance of *P. tomentosa*, reflected by decreased plant height, shoot and root dry weight, root-to-shoot ratio, total root length, root surface area, root volume, root-tip number, forks, and crossings (Figure 2 and Figure 3). By contrast, *R. irregularis* inoculation markedly decreased plant-growth inhibition under both As stress and non-As stress conditions. Under As stress, compared with non-inoculated CK100, Ri100 seedlings displayed 30.79% higher plant height, 145.71% elevated root dry weight, alongside substantial improvements across almost all measured root morphological parameters. These findings implied that *R. irregularis* colonization alleviated As-induced growth inhibition and promoted root system expansion in poplar seedlings. This AMF-mediated physiological protection was consistent with previous findings in host plants including *Robinia pseudoacacia*, cotton, and poplars exposed to heavy-metal and metalloid contamination [2,46]. AMF symbiosis enhanced host plant tolerance to abiotic stress by multiple interconnected physiological pathways, including improved acquisition of mineral nutrients (notably phosphorus), optimization of root system architecture, strengthened antioxidant defense capacity, and heavy-metal immobilization within root mycorrhizal structures [18,47]. The root-to-shoot ratio increase observed in Ri0 and Ri100 groups indicated that *R. irregularis* preferentially stimulated root biomass of *P. tomentosa*. Optimized root morphology (greater total root length, surface area, volume, branching points, and root-tip abundance) expanded the soil-exploration surface, enhanced absorption of nutrients in rhizosphere soil and mitigated toxic-ion-induced root-growth damage [48]. When comparing Ri100 versus Ri0, As stress still caused measurable declines in all traits of growth and root morphology, indicating that AMF inoculation alleviated but could not fully eliminate As-caused phytotoxicity [49].

Two-way ANOVA results provided statistical evidence for independent and interactive influences of As stress and *R. irregularis* inoculation on phenotypic traits of *P. tomentosa* (Table 1). Most biomass and root morphology parameters showed non-significant interaction between the two experimental factors. This statistical pattern suggested that for most vegetative growth traits, the positive effect of *R. irregularis* operated additively, rather than synergistically under As stress conditions [50]. Significant interaction for root surface area implied that As stress altered the magnitude by which AMF regulated root-surface expansion, and As directly damaged epidermal and cortical root tissues where AMF hyphae colonized [51]. Similar two-way ANOVA outcomes were documented in AMF-host plants-As interaction experiments in herbaceous crop systems, where heavy-metalloid stress and AMF inoculation frequently displayed significant interaction only for some physiological indicators [7]. These results provided experimental evidence that AMF alleviated As phytotoxicity in *P. tomentosa* seedlings by improving root system architecture and promoting seedling growth.

### 4.3. Functional Annotation and Enrichment of DEGs in P. tomentosa Induced by R. irregularis Inoculation and As Stress

Transcriptomics technologies with rhizosphere microbiome research revealed a bidirectional regulatory network mediated by root secretions in the host plant–AMF symbiosis [52]. In this study, GO and KEGG functional annotation for CK100 vs. Ri100 and CK0 vs. Ri0 illuminated major functional modules activated by *R. irregularis* inoculation and As stress (Figure 5), and comparing the DEG sets characterized the transcriptomic differences in *P. tomentosa* seedlings in response to *R. irregularis* inoculation under As stress conditions (Figure 5 and Figure 6). *R. irregularis* inoculation produced 3406 DEGs (1581 upregulated and 1825 downregulated; CK0 vs. Ri0) under non-As stress conditions, and generated 1186 DEGs (541 upregulated and 645 downregulated; CK100 vs. Ri100) under As stress, indicating that As stress changed the transcriptional landscape of *P. tomentosa* seedlings upon AMF symbiosis. Previous studies in woody plants under salt stress documented comparable numbers of enriched GO terms, highlighting the conserved nature of AMF-induced transcriptomic reprogramming [46]. The sharp reduction in total DEG numbers in Ri100 compared with Ri0 indicated that pre-existing As-stress-activated transcriptional programs constrained the magnitude of symbiosis-driven transcriptomic changes in *P. tomentosa* seedlings. Massive-scale transcriptional rewiring was also observed in mycorrhizal plant-stress transcriptome investigations [53]. Host plants need to balance two distinct signal inputs, including activation of the AMF symbiotic program and defense responses triggered by metalloid toxicity, to adapt to this dual-signal environment; numerous genes reverse their regulatory patterns [54].

KEGG enrichment for CK100 vs. Ri100 showed phenylpropanoid biosynthesis as the top-ranked enriched pathway (Figure 6D). Glutathione-based detoxification constituted a central cellular defense machinery for As-exposed plant cells, participating in As chelation, conjugation, and vacuolar compartmentalization [55]. Phenylpropanoid–lignin-pathway activation reinforced cell-wall rigidity, which helped root tissues counter As-provoked oxidative damage and cell-wall degradation [38,56]. In contrast, under non-As stress conditions (CK0 vs. Ri0), plant hormone signal transduction, plant–pathogen interaction, and MAPK-signaling plant pathways dominated KEGG enrichment outputs (Figure 6C). The above pathways represented canonical symbiotic signal perception modules for host roots, improving AMF signals and tuning plant defense responses to permit colonization [57]. The clear shift in dominant enriched pathway signatures between Ri0 and Ri100 comparisons demonstrated that As stress regulated the transcriptomic priorities of mycorrhizal *P. tomentosa* seedings from primarily symbiotic signal adjustment towards As detoxification and cell-wall reinforcement [58].

### 4.4. Expression Patterns of DEGs in P. tomentosa Related to Jasmonic Acid Synthesis and Metabolism Induced by R. irregularis Inoculation and As Stress

Plant jasmonic acid (JA) phytohormone signaling acted as a critical molecular hub integrating AMF symbiotic signaling and abiotic stress responses [32]. JA accumulation also facilitated early-stage AMF recognition and arbuscule formation; however, excessive JA activation triggered plant defense reactions that suppressed AMF colonization [59,60]. In *Citrus sinensis*, mycorrhizal symbiosis differentially regulated multiple JA-related genes under drought stress. However, the specific regulation of JOX, AOS, and AOC family genes by AMF in woody plants under As stress remains poorly understood [61].

Our transcriptome analysis characterized expression dynamics for 18 JA biosynthesis and metabolism-related genes belonging to “JOX, AOS, AOC, and MJE” (Figure 7). Under non-As stress, *R. irregularis* inoculation upregulated *JOX2-1*, *JOX2-3*, *JOX2-4*, *AOS2*, and *AOS7*, while repressing *AOC1* and *AOC2*, suggesting a basal modulation of JA metabolism that may facilitate symbiosis. Under As stress, AMF triggered more extensive reprogramming. *JOX2-1/2/3/4*, *JOX1*, *AOS6*, and *MJE1-1* were significantly induced, whereas *AOC1*, *AOC2*, *AOS2*, and *AOS7* were repressed. Notably, As stress alone suppressed most JOX and AOS genes, but activated *AOC1* and *AOC2* in non-inoculated roots, indicating stress-triggered JA accumulation that was partially reversed by AMF symbiosis. These findings suggested that *R. irregularis* inoculation balanced JA biosynthesis across multiple enzymatic steps. It promoted upstream oxygenation reactions, but restricted AOC-catalyzed conversion. This regulatory pattern optimized stress signaling and avoided excessive JA accumulation, which in turn improved arsenic tolerance in *P. tomentosa* seedlings.

### 4.5. Regulation of Transcription Factors by R. irregularis Inoculation and As Stress

Transcription factors served as central molecular switches that rewire downstream gene expression cascades during plant symbiosis and abiotic stress [62]. In this study, some differentially expressed TFs were identified in *P. tomentosa* induced by *R. irregularis* inoculation and As stress, with prominent representation from ERF, MYB, WRKY, bHLH, GRAS, and GATA families (Figure 9). In the Ri100 versus CK100 comparison, *R. irregularis* inoculation induced abundant ERF, GATA, WRKY, and NAC family members, whereas certain MYB-related and bZIP transcripts were repressed. In the Ri0 versus CK0 comparison, ERF, GATA, MYB-related, and NAC TFs were predominantly upregulated. Many of these TF families were established regulators for either AMF symbiosis or heavy metal stress tolerance in woody-plant species [21]. For example, ERF TFs mediated crosstalk among hormone signaling pathways and activated abiotic stress defense responses, WRKY and GATA TFs regulated defense-gene reprogramming in response to both microbial colonization and toxic-metal exposure, and NAC TFs modulated root development and oxidative-stress responses [58]. Notably, a large proportion of these TFs exhibited contrasting expression patterns in the presence or absence of As, indicating their dual involvement in AMF symbiosis and As stress signaling. Several candidate genes detected in our dataset, such as *GATA5*, *WRKY31*, and *ERF119*, were previously reported to participate in tree-species abiotic stress and symbiotic-regulatory networks [46].

To further elucidate the regulatory architecture, a weighted gene co-expression network (WGCNA) was constructed using the TFs and their associated genes (Figure 10). The final filtered network comprised 26 JA-biosynthetic-metabolic genes, 38 core TF nodes, and 94 significant interactions. Among the identified co-expression network, members of the GATA, WRKY, and MYB families, such as *GATA5*, *GATA16*, *WRKY57*, *MYB-PHL5*, and *MYB-PHL7*, displayed expression profiles highly consistent with those of the JA biosynthetic genes AOS and AOC. This coexpression pattern reveals correlative links between these transcription factors and JA-pathway gene expression under AMF-As stress conditions, which may connect mycorrhizal symbiotic signals with downstream hormonal defense responses. WRKY, MYB, and ERF TFs have been reported to modulate heavy-metal tolerance by activating antioxidant and cell-wall-related genes in other plant systems [63]. AMF colonization reprogrammed the expression of these TF families under salinity or metal stress in multiple plant species. For instance, WRKY6 in *Robinia pseudoacacia* was shown to repress As uptake by downregulating the phosphate transporter *PHT1;1*, thereby enhancing As tolerance. GATA5 showed tight co-expression with multiple JA-pathway genes in our dataset, suggesting it may participate in coordinating downstream stress-responsive processes linked to AMF symbiosis and arsenic stress. Extensive cross-regulation among these modules further revealed a multi-tiered transcriptional framework underpinning *R. irregularis*-mediated As tolerance in *P. tomentosa* seedlings. Overall, these results demonstrated that *R. irregularis* inoculation globally rewires the TF regulatory network in *P. tomentosa* seedlings, with *GATA5* and *WRKY57* serving as critical hubs that regulated JA signaling and multifaceted defense pathways to alleviate As toxicity. Besides JA-dependent signaling, other symbiosis-related regulatory modules may also participate in AMF-alleviated arsenic stress responses in *P. tomentosa*. GRAS-family transcription factors including NSP1, NSP2, RAM1 and DELLA are core components governing arbuscular mycorrhizal symbiosis. In particular, the NSP2-MYB complex has been demonstrated to modulate phenylpropanoid and flavonoid biosynthesis during mycorrhizal establishment. Whether these GRAS-MYB-related cascades crosstalk with JA signaling under arsenic stress remains unclear and merits further experimental investigation in future work.

## 5. Conclusions

The present study systematically characterized physiological, morphological and genome-wide transcriptomic responses of *P. tomentosa* to *R. irregularis* inoculation under As stress. As stress reduced mycorrhizal colonization, severely repressed seedling growth, and damaged root morphological architecture, whereas *R. irregularis* inoculation in *P. tomentosa* roots substantially ameliorated As-suppressed seedling growth and optimized root system architecture. Transcriptome analysis revealed massive transcriptomic reprogramming upon combined AMF symbiosis and As exposure. AMF symbiosis dynamically rewrote the transcriptional patterns of core JA biosynthesis and metabolism genes in *P. tomentosa* seedlings under As stress. A set of critical stress-responsive-TF families (ERF, GATA, WRKY, NAC, MYB, and bZIP) were differentially regulated by AMF symbiosis and As exposure. Weighted gene co-expression network analysis (WGCNA) highlighted hub TFs of the GATA and WRKY families (e.g., *GATA5* and *WRKY57*) as high-rank regulators governing JA-pathway gene expression. These TFs formed tight regulatory modules with core JA biosynthesis- and metabolism-related genes and were induced by *R. irregularis* inoculation and As stress. Our findings provided a theoretical mechanism for further elucidation of the JA signaling pathway of woody tree species in AMF-mediated alleviation of As toxicity, supporting future tree-breeding and phytoremediation technology development for As-polluted environments.

## Figures and Tables

**Table 1:** Effects of As stress, *Rhizophagus irregularis* inoculation, and their interaction on the growth of *Populus tomentosa*.

Parameters	As *p*-Values	AMF *p*-Values	AMF × As *p*-Values
Mycorrhizal infection rate	0.0036	0.0001	0.0001
Root dry weight	0.0077	0.0035	0.2821
Plant height	0.0068	0.0001	0.1738
Total root absorption area	0.0001	0.0185	0.2655
Root length	0.0018	0.0055	0.7327
Root area	0.0042	0.0001	0.0076
Root diameter	0.0061	0.0178	0.0185
Root volume	0.0001	0.0005	0.1497
Number of root tips	0.0066	0.0002	0.0549
Number of forks	0.0001	0.0264	0.192
Number of crossings	0.0001	0.0409	0.2167

## Data Availability

The raw RNA-seq data are available via NCBI SRA under BioProject PRJNA1252924. Other supporting data generated in this study are presented within the article and Supplementary Materials. Further inquiries can be directed to the corresponding author.

## Supplementary Materials

The following supporting information can be downloaded at https://www.mdpi.com/article/10.3390/biology15181653/s1. Figure S1: Schematic diagram showing the experimental design, cultivation, sampling and bioinformatics analysis workflow; Table S1: Transcriptome sequencing statistics and annotation data of expressed genes and transcripts; Table S2: Annotation analysis of expressed genes and transcripts in six databases.
