# Supplementary material for: Transcriptome Analysis of Populus tomentosa Reveals Molecular Mechanisms of Jasmonate Signaling Pathway on Arsenic Tolerance Induced by Arbuscular Mycorrhizal Fungi
Source: Biology (Basel). 2026 Sep 18;15(18):1653. doi: 10.3390/biology15181653 (PMC13604661; doi:10.3390/biology15181653)
Supplement: Supplementary file 1 [file biology-15-01653-s001.zip › biology-4548591-supplementary.pdf]

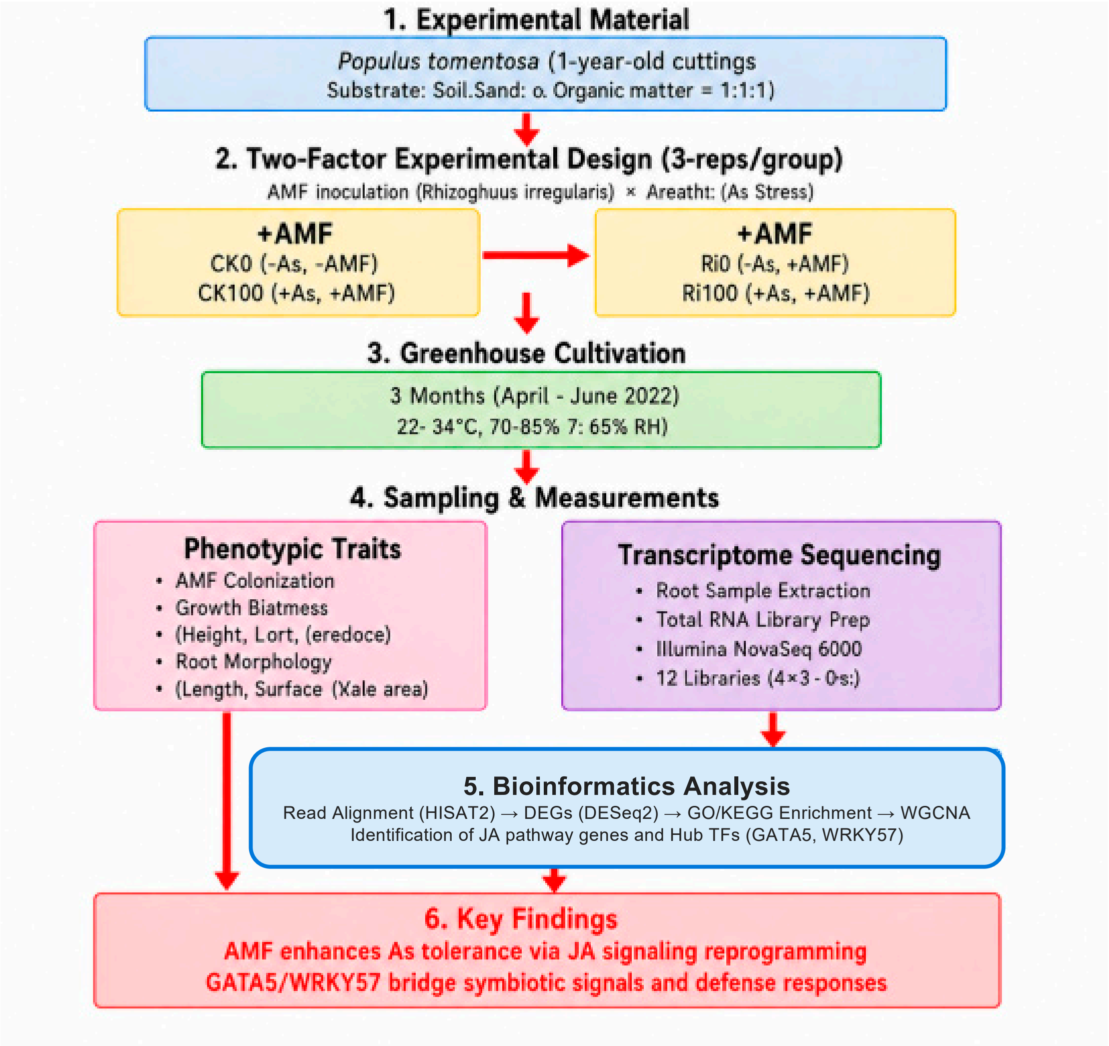

Figure S1. Schematic diagram showing the experimental design, cultivation, sampling and bioinformatics analysis workflow

Table S1. Transcriptome sequencing statistics and annotation data of expressed genes and transcripts

Sample	Raw reads	Clean reads	Clean reads ratio (%)	Q20%	Q30%	GC (%)	Total reads	Total mapped	Multiple mapped	Uniquely mapped
CK0_1	43798860	43395622	98.74	98.39	94.97	43.68	43395622	34333189(79.12%)	899556(2.07%)	33433633(77.04%)
CK0_2	44665544	44254660	98.75	98.46	95.17	43.54	44254660	35817023(80.93%)	1064434(2.41%)	34752589(78.53%)
CK0_3	43383710	42986924	98.75	98.44	95.13	43.68	42986924	34725983(80.78%)	1274467(2.96%)	33451516(77.82%)
CK100_1	40806334	40421646	98.74	98.38	94.93	43.8	40421646	32679651(80.85%)	966019(2.39%)	31713632(78.46%)
C100_2	46240862	45834022	98.74	98.39	94.99	43.65	45834022	37560663(81.95%)	977018(2.13%)	36583645(79.82%)
CK100_3	42201794	41818464	98.74	98.4	95.02	43.62	41818464	33840937(80.92%)	939491(2.25%)	32901446(78.68%)
Ri0_1	40909480	40526464	98.74	98.4	95.01	43.81	40526464	33140959(81.78%)	1218999(3.01%)	31921960(78.77%)
Ri0_2	47300264	46835000	98.74	98.38	94.96	43.69	46835000	38043185(81.23%)	1069099(2.28%)	36974086(78.95%)
Ri0_3	44999256	44574682	98.75	98.43	95.1	43.77	44574682	36668851(82.26%)	1206522(2.71%)	35462329(79.56%)
Ri100_1	48598564	47481844	98.75	98.45	95.14	43.84	45140120	35845710(79.41%)	1111825(2.46%)	34733885(76.95%)
Ri100_2	45445792	45140726	98.77	98.53	95.42	44.09	46098744	36608373(79.41%)	1160388(2.52%)	35447985(76.9%)
Ri100_3	46604696	46136540	98.76	98.51	95.33	43.81	45221740	37414219(82.74%)	1156125(2.56%)	36258094(80.18%)

Table S2. Annotation analysis of expressed genes and transcripts in six databases

Database Name	Expressed Gene number （percent）	Expressed Transcript number （percent）	All Gene number （percent）	All Transcript number （percent）
GO	24748(0.8084)	41390(0.7967)	27211(0.7890)	48210(0.7821)
KEGG	14803(0.4836)	25433(0.4895)	15702(0.4536)	29098(0.4720)
COG	28991(0.947)	49564(0.9540)	31323(0.9048)	57214(0.9282)
NR	30348(0.9914)	51664(0.9944)	33892(0.9790)	60851(0.9872)
Swiss-Prot	25383(0.8292)	43442(0.8361)	27173(0.7849)	49809(0.8080)
Pfam	25084(0.8194)	41049(0.7901)	26491(0.7652)	46245(0.7502)
Total annotation	30350(0.9914)	51667(0.9945)	33897(0.9792)	60862(0.9873)
Total	30612(1.0000)	51955(1.0000)	34618(1.0000)	61642(1.0000)
